# Footprints of Sepsis Framed Within Community Acquired Pneumonia in the Blood Transcriptome

**DOI:** 10.3389/fimmu.2018.01620

**Published:** 2018-07-17

**Authors:** Lydia Hopp, Henry Loeffler-Wirth, Lilit Nersisyan, Arsen Arakelyan, Hans Binder

**Affiliations:** ^1^Interdisciplinary Centre for Bioinformatics, Universität Leipzig, Leipzig, Germany; ^2^Group of Bioinformatics, Institute of Molecular Biology, National Academy of Sciences, Yerevan, Armenia

**Keywords:** immune suppression, epigenetics, infections, blood disturbances, community-acquired pneumonia severity, molecular subtypes, prognostic impact

## Abstract

We analyzed the blood transcriptome of sepsis framed within community-acquired pneumonia (CAP) and characterized its molecular and cellular heterogeneity in terms of functional modules of co-regulated genes with impact for the underlying pathophysiological mechanisms. Our results showed that CAP severity is associated with immune suppression owing to T-cell exhaustion and HLA and chemokine receptor deactivation, endotoxin tolerance, macrophage polarization, and metabolic conversion from oxidative phosphorylation to glycolysis. We also found footprints of host’s response to viruses and bacteria, altered levels of mRNA from erythrocytes and platelets indicating coagulopathy that parallel severity of sepsis and survival. Finally, our data demonstrated chromatin re-modeling associated with extensive transcriptional deregulation of chromatin modifying enzymes, which suggests the extensive changes of DNA methylation with potential impact for marker selection and functional characterization. Based on the molecular footprints identified, we propose a novel stratification of CAP cases into six groups differing in the transcriptomic scores of CAP severity, interferon response, and erythrocyte mRNA expression with impact for prognosis. Our analysis increases the resolution of transcriptomic footprints of CAP and reveals opportunities for selecting sets of transcriptomic markers with impact for translation of omics research in terms of patient stratification schemes and sets of signature genes.

## Introduction

Community-acquired pneumonia (CAP) is one of the most common infectious diseases, and it is an important cause of mortality and morbidity worldwide (1). CAP is an acute infection of the pulmonary parenchyma with symptom onset in the community. A complication of CAP is severe sepsis, the syndrome of infection often accompanied by systemic inflammation and organ dysfunction. Assessment of CAP severity is a cornerstone in its management, facilitating selection of the most appropriate site of care and empirical antibiotic therapy (2). Although a series of scores have been developed such as the Pneumonia Severity Index and Sequential Organ Failure Assessment (SOFA) score, so far, no ideal solution has been identified (3, 4). Increasing attention has been paid to research on biomarkers, since they have the potential to resolve fundamental issues regarding prognostic prediction that cannot be readily addressed using CAP-specific scores (5, 6). Biomarker selection, severity assessment, and therapy require an understanding of the heterogeneity of the individual host response to infection and of the underlying molecular determinants.

Whole genome transcriptomic profiling of tissue-biopsies has been shown to successfully address these objectives in a large number of applications dealing with different diseases, such as cancer, infectious and civilization diseases (7). On the other hand, human peripheral blood can be understood as surrogate “liquid” biopsy material providing a less invasive option for patients. Its transcriptional profiling might reflect physiological and pathological events occurring in different tissues of the body (8). Its application is, however, potentially hampered by a series of factors such as the complexity of processes contributing to the blood transcriptome and also its stability. Only recently, a few interesting studies on the blood transcriptome of sepsis framed within CAP have been published that provided novel insights into alterations of gene expression (GE) caused by this disease and also into underlying physiological processes such as immune suppression and endotoxin tolerance (ET) in severe CAP cases (9–11).

Previously, we have developed an omics “portrayal” method based on machine learning using self-organizing maps (SOM) that enables a very detailed evaluation of the landscapes of transcriptomic data (12, 13). It takes into account the multidimensional nature of gene regulation and pursues a modular view on GE, reduces dimensionality, and supports visual perception in terms of individual, case-specific “omics” portraits. The method was applied to a series of data types and diseases (13–19). In the first part of this publication, we re-analyzed the published CAP blood transcriptomic data (peripheral blood leukocytes) (9) using this method in order to disentangle the molecular heterogeneity of CAP with high resolution, to generate sample and feature landscapes, and to extract its modular structure, which is a straightforward concept, as has been demonstrated before for the healthy blood transcriptome (20). We aim at characterizing transcriptomic footprints of the immune response, of selected blood cell lineages (erythrocytes, platelets, T-cells, and macrophages), and of host’s response to viral and bacterial infections. Special attention is paid toward footprints of epigenetic changes in the blood transcriptome of CAP cases, a so-far, weakly studied field, although its importance for the development of infectious diseases becomes increasingly accepted (21). In the second part of the paper, we propose a novel stratification scheme of CAP based on the multidimensional structure of the blood transcriptome as seen by our analysis together with a glimpse on the dynamics of transcriptomic changes in the first days of the disease and on the prognostic impact of the new classes.

We, hereby, pursue a multi-omics systems-based approach that is expected to enhance our understanding of the biology of sepsis framed within CAP and supports the translation of omics research in terms of stratification of CAP as a heterogeneous disease into subtypes and of extracting sets of signature genes with possible diagnostic and/or prognostic impact (22).

## Materials and Methods

### Patients and GE Data

We have re-analyzed transcriptomic data of blood samples of CAP patients as described in Ref. (9, 10). CAP was defined as a febrile illness associated with cough, sputum production, breathlessness, leukocytosis, and radiological features of pneumonia acquired in the community or within 2 days of intensive care unit (ICU) admission. The cohort was recruited concurrently from 25 ICU in the UK during 8 years (2006–2014) and processed in parallel and independently as a discovery and a prospective validation cohort where equal numbers of survivors and non-survivors were selected in the validation cohort for optimal contrast as described in Ref. (9). GE was investigated in peripheral blood leukocytes and compared between CAP patients and 10 subjects undergoing elective cardiac surgery serving as control. Preprocessed GE data (Illumina HumanHT-12 v4 platform) used in our study was taken from the publication of Burnham et al. (10) who compared transcriptomes of sepsis caused by CAP and fecal peritonitis (FP). Patients diagnosed as FP were not included in our analyses. CAP data were split in two cohorts: the discovery cohort consists of 127 samples taken from 73 patients at 1, 3, and 5 days after ICU admission [see Figure S1 in Supplementary Material for an overview and also Table 1 in Ref. (10) for patients description]. Some individuals from discovery cohort were sampled at several time points. The validation cohort consisted of 53 samples taken from 53 patients after ICU admission. These data were part of a larger study by Davenport et al. (9). In addition to the original labeling, we applied a sorting of samples using a “severity”-score and, finally, we propose a novel stratification of samples based on the multidimensional structure extracted from the transcriptome data.

### Expression Portrayal Using SOM

Transcriptome analysis was performed using the omics “portrayal” method that was developed by our group based on machine learning using SOM (12, 13, 23). It enables a detailed evaluation of the landscapes of transcriptomic data, takes into account the multidimensional nature of gene regulation, pursues a modular view on GE, and supports a visual perception guided analysis. SOM portrayal itself represents a dimension-reduction approach that keeps the intrinsic data structure unchanged and unaffected by the class labels of the samples. We have combined discovery and validation cohorts and used initial grouping of samples proposed in Ref. (10) in order to obtain a global view on their transcriptomic landscapes as well to disentangle any systematic differences between cohorts and/or the original classification groups. With this aim, we trained one SOM using the whole CAP-related sample set in order to obtain a holistic view of all relevant transcriptional modes inherent in the data. Note also that comparison of the “personalized” expression portraits require a joint SOM training to make the portraits mutually comparable. For this, gene-centric expression data were quantile normalized, then centralized and clustered using the SOM machine learning algorithm. The SOM-portrayal method translates the gene-centric data profiles into 2,500 metagene profiles and visualizes their levels using a two-dimensional quadratic 50 × 50 pixel map and a maroon-to-blue color code for high-to-low metagene expression values (12). The mosaic image obtained for each sample serves as a fingerprint portrait of its GE landscape (Figure S2 in Supplementary Material). Class-specific mean portraits were generated by averaging the metagene landscapes of all cases belonging to one class. Difference portraits between them were calculated as the differences between the metagene values in each pixel of these maps.

A “prognostic map” was generated by associating survival data (28-day survival) with the expression levels of the metagenes in their SOM-portraits as follows: the survival rate of all cases showing expression levels exceeding the SD of expression of the metagenes was counted as the percentage of survivors referring to this particular metagene, which is then color coded in the map. Metagenes providing less than 10% of cases were not considered for the survival analysis (white areas in the map).

### Bioinformatics Analysis

Downstream bioinformatics analyses were performed based on the SOM portraits as described previously (12, 23). This includes diversity analysis of the sample’s SOM, feature selection in terms of so-called spot clusters, and functional interpretation in terms of gene set analysis. Diversity analysis was performed using a graph representation called correlation network and pairwise correlation maps. Clusters of co-expressed genes were obtained by segmentation of the SOM images using the “maximum-distance between neighboring pixels” (U-map)-topology as implemented in Ref. (24). For functional interpretation, we applied gene set analysis by means of spot enrichment using Fishers exact test, of gene set enrichment *Z*-score (GSZ) profiling and of population maps of the set genes that show their positions in the SOM (25). Gene sets were taken from GSEA-repository (26) using the categories gene ontology (GO) “biological process” and “hallmarks of cancer” (23, 26, 27), from a previous study on the blood transcriptome (20) and from the literature for different other functional categories (Table S1 in Supplementary Material). Chromatin state-related gene sets for selected blood compounds were extracted from the respective genomic regions, which were taken from a recent study of the NIH Roadmap Epigenomics Consortium for a series of epigenetic states (28). We assigned all genes within these regions to the respective state and further processed them in analogy to gene sets that provided GSZ-values for each sample [see also Ref. (29) for details]. Heatmap presentations were generated by standard R-functions including hierarchical clustering options. The activity of selected KEGG-pathways was estimated using the pathway signal flow (PSF) method (30). All the methods were implemented in the R-package “oposSOM” used for the analysis (24).

### Class Discovery

Class discovery of new CAP classes was performed by K-means clustering based on Pearson’s correlations coefficients (“*r*”) of all pairwise combinations of SOM portraits. The class stability “silhouette” score “*S*” was calculated for each sample as a quality estimate how it fits into its class compared with the other classes (19, 29). The score is defined as the difference between the difference of the *r*-value of each sample and its cluster centroid and the smallest (i.e., the best) difference to any centroid’s *r*-value of the other classes. The sample similarity score is positive if the sample best fits into its class while it is negative if another class provides closer similarity. The result is presented as silhouette plot that shows the *S*-ranked samples for each class. After initial *K*-means clustering the *S*-score is maximized by iteratively assigning the samples to best-matching classes to increase their *S*-score until convergence. Cluster stability is estimated by bootstrapping and calculating the mean percentage of samples that maintain their class label.

## Results

### The Landscape of the CAP Blood Transcriptome

**Figure 1:**
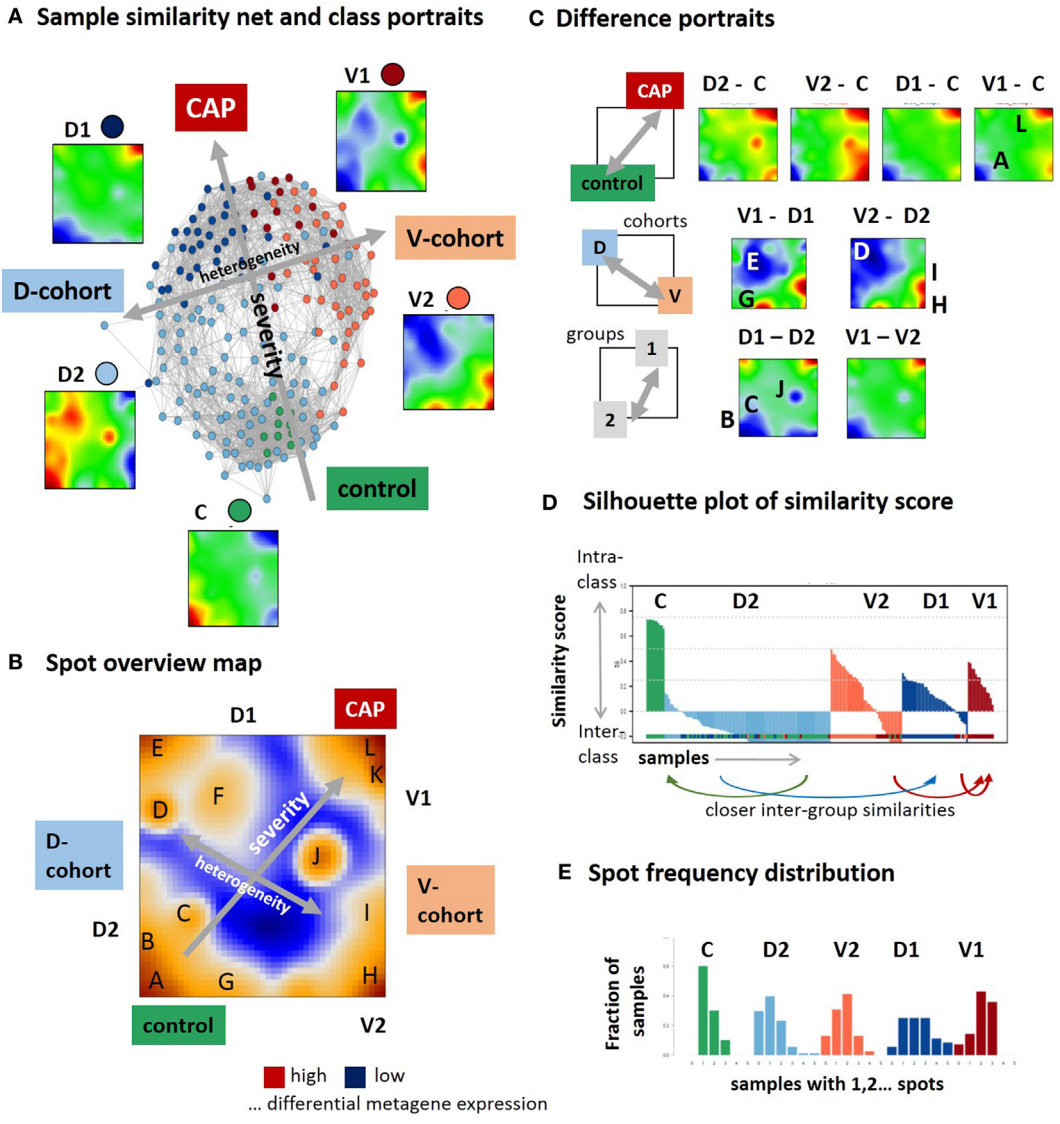
Self organizing map (SOM) portrayal of the blood transcriptome of community-acquired pneumonia (CAP). **(A)** The sample similarity (correlation) network and the mean expression portraits of the five sample groups, healthy control **(C)**, discovery cohort groups 1 and 2 (D1 and D2), and validation cohort group 1 and 2 (V1 and V2) reveal two major dimensions of diversity (arrows). **(B)** The expression variance map indicates 12 major spots (A–L) of co-expressed genes. **(C)** Difference portraits between the sample groups reveal modes of differential expression. **(D)** The similarity score describes the similarity of each sample to its own cluster. Negative scores indicate that the sample is more similar to other clusters. The figure shows that D2 is an unspecific group for which most of the samples are more similar to C and D1 (see arrows) while C and V1 are specific groups. **(E)** The spot frequency distributions indicates that sample SOM portraits express more spots in direction from the left to the right, thus reflecting a larger heterogeneity of transcriptional programs activated in CAP compared with the controls.

**Figure 2:**
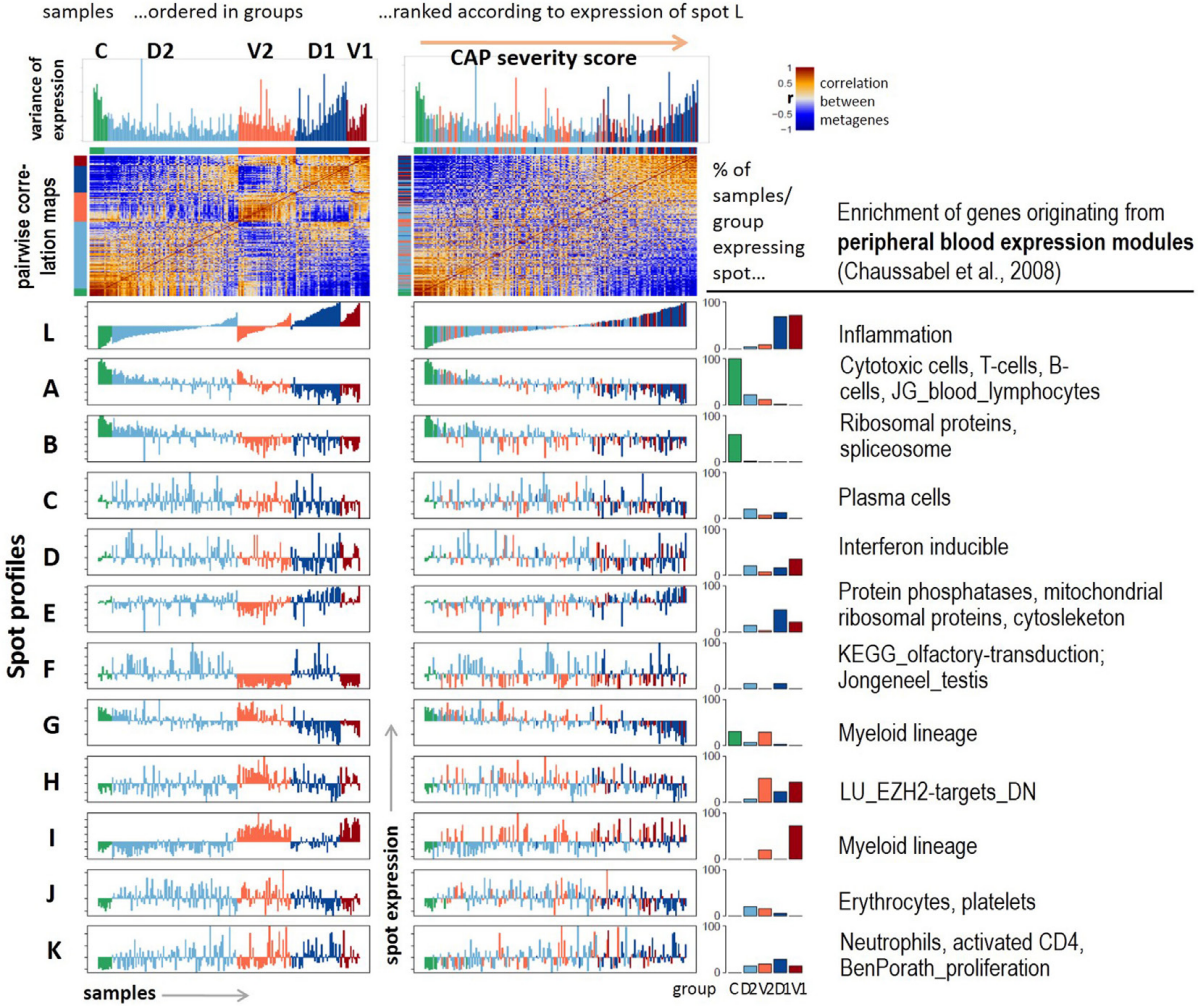
Profiling of the expression modules: pairwise correlation maps of the sample self organizing maps and of the expression profiles of the spot modules were generated in two variants of sample sorting using either the group-classification (left part) or the inflammatory activity as estimated by the mean gene expression of spot L (right part). The barplots in the right part show the percentage of samples of each class expressing the respective spot. Healthy blood signatures enriched in the spots (*p* < 10^−7^, Fishers exact test) were taken from Ref. (20, 31) and other signatures from Ref. (32, 33).

In the first step, we have generated SOM transcriptomic portraits of all samples studied (Figure S2A in Supplementary Material). Then, we have calculated the mean portraits of each group considered and a similarity network for an overview of transcriptional diversity of the samples (Figure 1A). In this network, the samples are roughly spread along two major dimensions, (i) directing either from the healthy controls (C) toward group 1 samples of the discovery (D1) and validation (V1) cohorts or (ii) dividing the discovery and validation cohorts (see arrows in Figure 1A). The mean group portraits show specific over- and underexpression patterns as red and blue spot-like areas in different regions of the maps. The spot areas collect co-expressed genes with mutually correlated expression profiles that concertedly show high or low expression values in the different sample groups in terms of the over- and underexpression spots mentioned (the profiles were shown in Figure 2 below). For an overview, we make use of the variance map of GE that reveals the spot-clusters of co-expressed genes as brown areas of highly variant expression (Figure 1B). In total, we identified 12 major spots labeled by capital letters “A–L” (gene lists are provided in Table S2 in the Supplementary Material). The spot-activation patterns of the groups reflect symmetry along two major dimensions similar to that of the samples (see arrows in Figure 1B). Difference portraits between the groups further support this result (Figure 1C): The two spots A and L antagonistically switch between all CAP- and the control groups, thus collecting genes commonly down and upregulated in CAP compared with the controls. Therefore, they refer to the major dimension on GE level. Contrarily, both cohorts differ systematically by spot activation in the left and right part of the map.

The stability of classification was estimated by means of the silhouette plot in Figure 1D (17). It reflects high stability for C-samples by positive values while D2-samples show closer similarities either with C- or D1-groups as indicated by negative values of the similarity score. Note that our similarity analysis is based on the whole transcriptome expression portraits and thus it does not contradict the classification taken from the original publications (9, 10). It, however, demonstrates that group D2 collects samples that form a continuum of transcriptional states linking the healthy blood transcriptome in C and the CAP transcriptome in D1. The results indicate the heterogeneity in the discovery and validation cohort samples but also systematic differences between them. While the molecular portraits of D1 and V1 show similar spot distribution, V2 and D2 are clearly different indicating the incompatibility of group 2 samples across cohorts. The mean SOFA-score at the day of sampling averaged over all samples of each group in group 1 exceeds that of group 2 [7.9 ± 4 versus 5.4 ± 3.2 in the D-cohort and 8.6 ± 4.9 versus 6.7 ± 3.2 in the V-cohort, see Table 2 in Ref. (9)]. Moreover, the mortality (after 14 days, 28 days, and 6 months) is significantly higher in group 1 compared with group 2 in the D- and V-cohorts as well [*p* < 0.04, 14-day mortality 22% for group 1 versus 10% for group2, hazard ratio 2.4, see Table 2 in Ref. (9)]. Thus, on the average, group 2 collects samples of a less severe form of CAP compared with group 1. Hence, the major dimension, which directs from controls *via* group 2 to group 1 can be interpreted as the major severity axis of CAP in this data. The closer similarity of C-samples with D2 can be explained by the fact that C-samples were collected as part of the D-cohort (9). The number of overexpression spots detected in each of the samples were summarized in the spot-number distribution shown in Figure 1E. C-group samples mostly overexpress only spot A, while the number of spots progressively increases in group 2 and even more in group 1 resulting in a broad distribution of spot numbers. This trend reflects an increased heterogeneity of activated transcriptional programs in CAP compared with healthy blood, which is further gained with CAP severity.

In summary, SOM portrayal resolves heterogeneity of samples and of transcriptional programs along two major dimensions describing either the severity of CAP or systematic differences between the two cohorts studied.

### Modules of Co-Expressed Genes

Next, we aimed at studying the spot expression characteristics more in detail. For this purpose, we ranked the samples in two different ways: first, according to their group memberships to highlight systematic differences between the groups (Figure 2, left part); and second, with increasing expression of spot L. It was assigned to upregulated expression in CAP versus control samples and to group 1 versus group 2 cases (Figure 1B). We, therefore, assume that this dimension roughly estimates the severity of CAP and, therefore, we used it as transcriptomics measure of CAP severity. Ranking along this axis associates with a *U*-shaped variability profile of GE, which reflects the fact that this dimension covers the major dimension of transcriptional variability. Independent component analysis (ICA) supports this result: the first component (IC1) describes the variability along the severity axis, while IC2 captures the variability introduced by the two cohorts (Figure S3 in Supplementary Material). ICA also shows that the variability of the transcriptomes due to independent processes gains along the severity axis in agreement with the spot-frequency distributions (Figure 1E). The pairwise similarity heatmap further divides the samples along this axis into three main categories, which either resemble the control group or the severe CAP cases in group 1, or an intermediate category forming a sort of continuum between these two bordering situations.

The functional context of each spot was characterized using gene set analysis based on expression signature genes from a previous study on healthy blood (20) and other literature sources (Figure 2, table in the right part and Figure S4 in Supplementary Material). The major alterations of GE due to CAP were assigned to a strong gain of inflammatory function (spot L) paralleled with the decay of T- and B-cell expression signatures (spot A). Both cohorts differ mainly in the expression of mitochondrial translation (spot E), G-protein coupled receptors (GPCR) and olfactory transduction (spot F), and functions related to myeloid lineage (spots G and I). Spots C (plasma cells), J (platelets, erythrocytes), and K (neutrophils) show rather scattered expression profiles that virtually seem to not fit into the two major dimensions of heterogeneity established above. Instead, they show maximum of expression in group 2 samples (spot J) or they reveal a weak increasing (spot K) or decreasing (spot C) trend, however, with strong sample-to-sample variability. The correlations between the spots are negative for the two main dimensions of heterogeneity, thus reflecting antagonistic changes between the respective genes (Figure S5 in Supplementary Material). The spots F (olfactory receptors) and J (platelets and erythrocytes) only weakly correlate with the spots of the two main dimensions.

Hence, the expression landscape decomposes into a set of one dozen spot modules of co-regulated genes of defined functional context and with different impact for CAP severity and heterogeneity.

### Immune Response and Disease Severity

**Figure 3:**
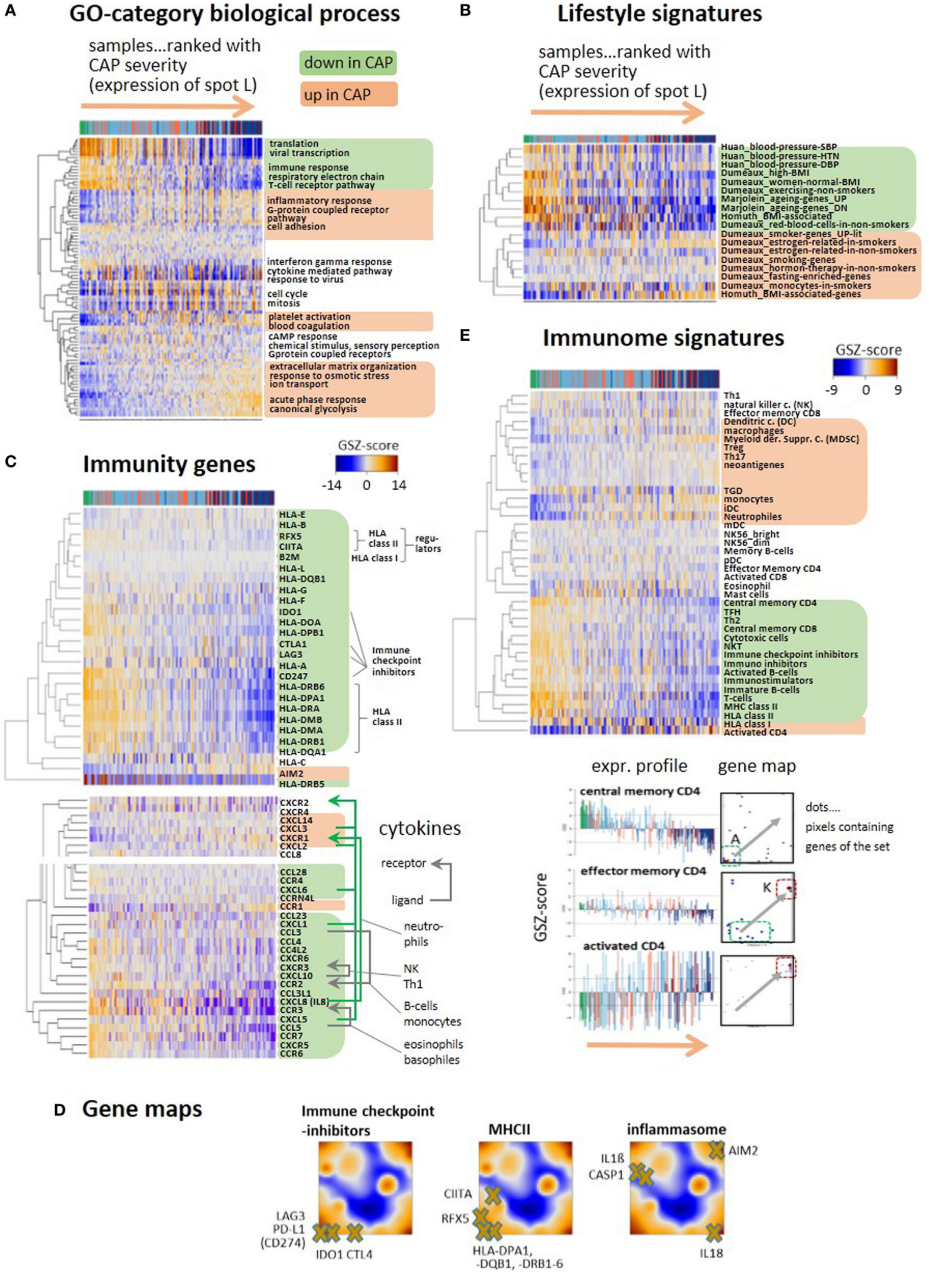
Gene expression heatmaps of functional categories **(A)** gene ontology biological process, **(B)** lifestyle, aging, BMI, and blood pressure signatures of peripheral blood taken from Ref. (35–38). **(C)** Selected genes with functions in immune response such as HLA type I and II, their regulators (*BLM2, CIITA, RFX5*), immune checkpoint inhibitors, and the inflammasome gene AIM2. All heatmaps reveal two major clusters of either decaying (green background) or increasing (apricot) expression with increasing community-acquired pneumonia (CAP) severity. Cytokines show similar profiles. Selected receptor–ligand pairs are connected by arrows and assigned to the respective immune cells according to Ref. (39). **(D)** Selected immunity genes were mapped onto the self organizing map portraits, where immune checkpoint inhibitors and MHC II-related genes accumulate in the region of spot A collecting genes that become deactivated in CAP. **(E)** GSZ-heatmaps “Immunome” signatures of immune cells and constituents, as provided by Ref. (40, 41). The part below shows gene maps and expression profiles of signature genes characterizing different stages of CD4 activation: upon activation the position of the respective genes shift from spot A to spot K (see gray arrows in the maps), where they show either decaying or increasing expression profiles.

We performed gene set expression analysis in terms of the gene set *Z*-score [GSZ (25)] using different functional categories. The expression scores of sets of the GO “Biological process” terms such as transcription, immune response, and T-cell receptor (TCR) pathway decrease, while that of inflammatory response, extracellular matrix organization, acute phase response, and glycolysis increase with disease severity (Figure 3A). Also, “lifestyle” (Figure 3B) and “hallmarks of cancer” (Figure S6 in Supplementary Material) signatures reveal each two groups of gene sets that up- and downregulate with disease severity. The latter functional category characterizes cell and pathway activities frequently dysregulated in cancer and other diseases. Only few sets such as “Myc-targets,” “DNA-repair,” and “oxidative phosphorylation” (oxphos) decay, while a larger number of sets mostly gain activity presumably due to their relation to inflammation (e.g., “TGFbeta,” “PI3K-Akt-MTOR,” and “IL6-JAK-Stat3”-signaling, “hypoxia,” “coagulation,” “reactive-oxygen-species”). Metabolic switching from oxphos to glycolysis was reported previously to constitute the metabolic basis of immunity due to the glucose consumption of trained monocytes (34). Similar changes were observed for gene signatures, which were related to obesity, smoking, blood pressure, and aging. This result suggests an overreaching responsiveness of the blood transcriptome to multiple factors in terms of the processes discussed here (Figure 3B). Moreover, part of the heterogeneity of the CAP-transcriptome potentially can originate from the heterogeneity of the blood transcriptome of the patients under healthy conditions before CAP onset.

Expression of a whole battery of chemokines, among them, *CXCL1, 5, 8* (*IL8*), and *10* is initially high, thus indicating stimulation of immune response; but it decreases with CAP severity, reflecting progressive immune suppression (Figure 3C). A series of receptor–ligand pairs show concerted profiles suggesting de-activation of the respective immune cells (39). Another subset of chemokines, *CXCL2, 3*, and *14* changes expression into the opposite direction and gets upregulated in CAP. The decay of immune response can be attributed to loss of HLA class II antigen presentation and to T-cell exhaustion, as indicated by the downregulation of GE of *HLA* class II antigens, of their regulator genes (*CIITA* and *RFX5*) and of the immune checkpoint inhibitors *CTL4, IDO1, LAG3*, and *PD-L1* in agreement with (9) (Figure 3C). In contrast, disease severity is paralleled by increasing expression of the inflammasome *AIM2* indicating activation of host response against cytosolic bacteria and viruses (42). Gene maps localize genes mentioned above within the expression landscape of CAP (Figure 3D). Immune checkpoint blockade genes and the MHC II compounds, all locate in or near spot A, thus reflecting their co-expression with the A-spot profile. In contrast, location of *AIM2* in spot L clearly indicates co-expression with the inflammatory characteristics of this spot. Genes affected by *AIM2* such as *CASP1, IL1-beta*, and *IL18* locate in different regions of the map presumably due to modulation of their expression by other factors, such as specific host response to viruses (see below).

A comprehensive collection of gene sets referred to as “immunome” (40, 41) supports these results and provides further details (Figure 3E): the cluster associated with decaying adaptive immune response includes signatures of lymphoid cells such as T-cells, central memory CD4 and CD8 cells, cytotoxic cells, and B-cells. Increasing inflammation associates with the gain of expression signatures of myeloid cells, such as monocytes, neutrophils, and myeloid-derived suppressor cells (MDSC), all with different roles in innate immunity (21). MDSCs are considered immunosuppressive cells, are increased in sepsis, and can block specific T cell function (43). A third group of gene sets upregulates in an intermediate range enriched with group 2 patients and includes the signatures of eosinophils and mast cells. The signature genes of central memory CD4 accumulate in and near spot A and that of activated CD4+ in and near spot K while that of effector memory CD4 reflect intermediate characteristics. These alterations illustrate differentiation of CD4 cells (44) and activation of inflammatory response in the course of CAP.

In summary, the major severity axis of CAP is characterized by progressive decay of immune responsiveness due to T-cell exhaustion and loss of MHC II activity paralleled by the gain of expression signatures of inflammation, myeloid blood compounds, glycolytic activity, and hypoxia.

### Footprints of Viral and Bacterial Infections

**Figure 4:**
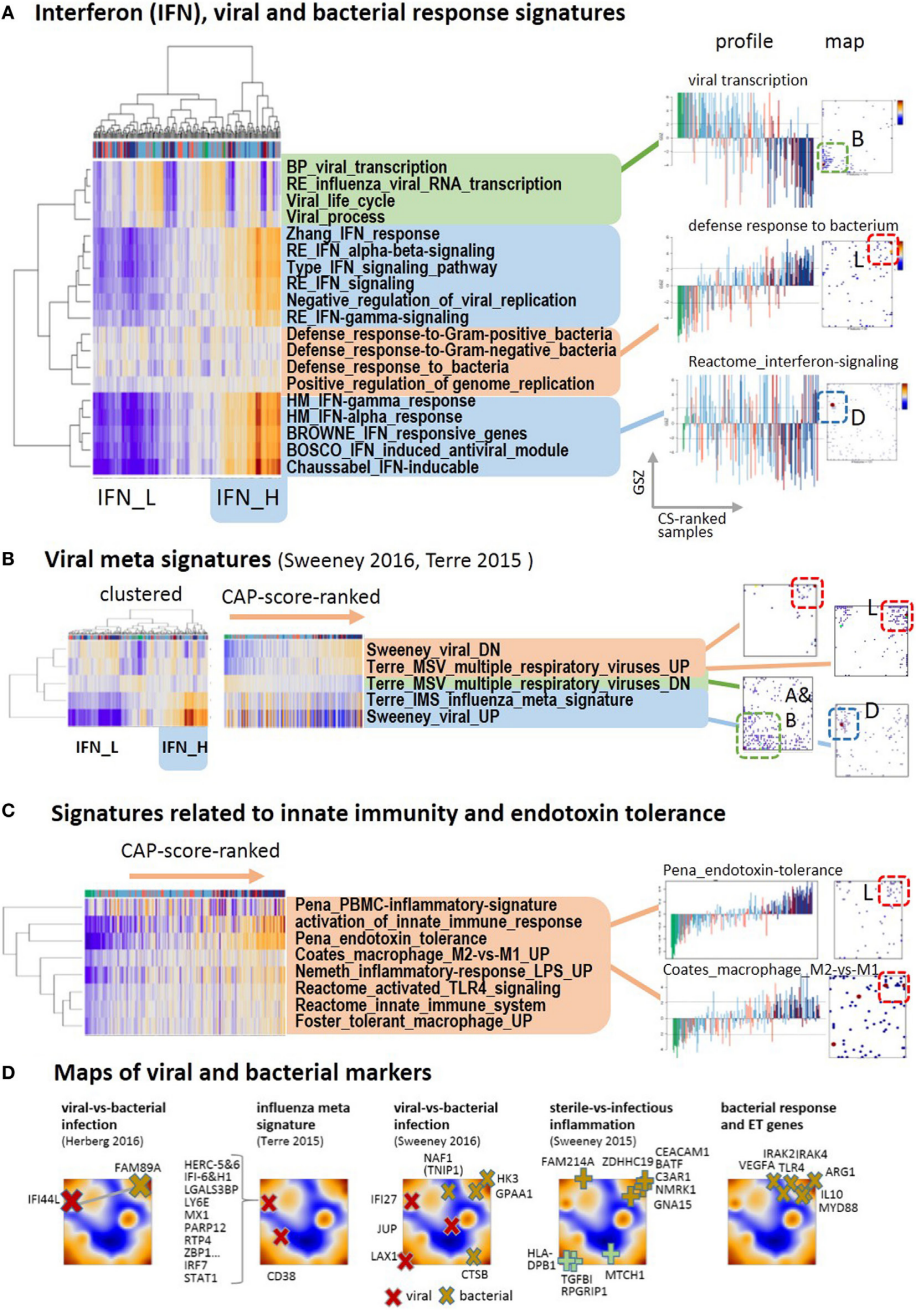
Footprints of infections in the community-acquired pneumonia (CAP) transcriptome: **(A)** two-way hierarchical clustering of interferon-related, bacterial and viral response signatures reveals two clusters of IFN-high (IFN-H) and IFN-low (IFN-L) responsive cases, in addition to clusters of cases with up and down regulated expression according to CAP severity. The IFN-responsive genes accumulate strongly in spot D. **(B)** Gene sets from recent meta-analyses (46, 47) split into the same clusters as the gene sets in part A. The IFN-responsiveness associates with the signature for viral infections. The respective genes accumulate in different characteristic spot areas of the map. **(C)** The expression of gene signatures related to bacterial infections and endotoxin tolerance taken from Ref. (48–51) increases with CAP severity. **(D)** Gene markers that differentiate between different types of infections taken from Ref. (46, 47, 52) are mapped into the expression self organizing map. They locate typically in/near spots D (viral markers), L (bacterial and ET markers), and A (sterile infection).

Respiratory viruses including influenza are considered as one of the causes of CAP, particularly among children and people with serious medical comorbidities (45). Co-infection with viruses and bacteria is also common, and it remains challenging to determine which patients have only viral or mixed infection as the cause of CAP. Using a series of standard gene sets related to viral and bacterial infections, we identified three clusters of functional signatures (Figure 4A). The first one (green background color) decays in expression along with increase in CAP severity: it refers to viral transcription and spot B. This cluster, however, lacks high specificity for viral infections because it seems to intermix with characteristics of the normal transcriptional machinery of the blood cells. The expression of the second cluster increases with CAP severity, it relates to the responses to bacterial infections and is associated with spot L (red background color). The third cluster collects a battery of gene sets characterizing interferon responses (blue background color). Their genes densely accumulate in spot D associated with very scattered profiles that relatively and weakly correlate with the major CAP severity dimension (see also below). The heatmap presentation in Figure 4A also reveals that clustering splits the samples roughly into two strata of high and low IFN-response with accumulation of samples from D2 in the former one and of D1 in the latter one.

Next, we analyzed recently published gene signatures derived from multi-cohort meta-analyses to differentiate between different types of infections (46, 47). Their expression patterns almost reproduce the three clusters mentioned above (Figure 4B): specific signatures for viral infections closely agree with the interferon-related signatures, whereas signatures of genes downregulated upon viral infections increase with CAP severity and resemble that of innate immune response and bacterial infections (see below Figures 4A,D). Further reduced sets of marker genes that were generated by several authors to robustly identify viral infections by overexpression commonly locate in spot D of interferon inducible genes (Figure 4D). For example, 10 out of the 11 genes of the influenza meta signature locate in spot D together with their transcriptional regulators *IRF7* and *STAT1* (46). Contrarily, marker genes of bacterial infections virtually upregulate with CAP severity as indicated by their location in and near spot L. A collection of key genes of bacterial response and ET, such as *TLR4, MyD88, IL10, IRAK2, IRAK4*, and *ARG1* (53, 54) also locate in and around this spot. This indicates their progressive activation with CAP severity together with activation of a series of gene sets related to bacterial response and ET (Figure 4C). The signatures of *TLR4*-pathway signaling, of ET (48), of macrophages, of an LPS-tolerant state (49), and of the polarization of macrophages to an immunosuppressive M2 state (50), which is shown to resemble ET macrophages (39, 53, 55), all suggest development of ET with CAP severity in agreement with (9).

**Figure 5:**
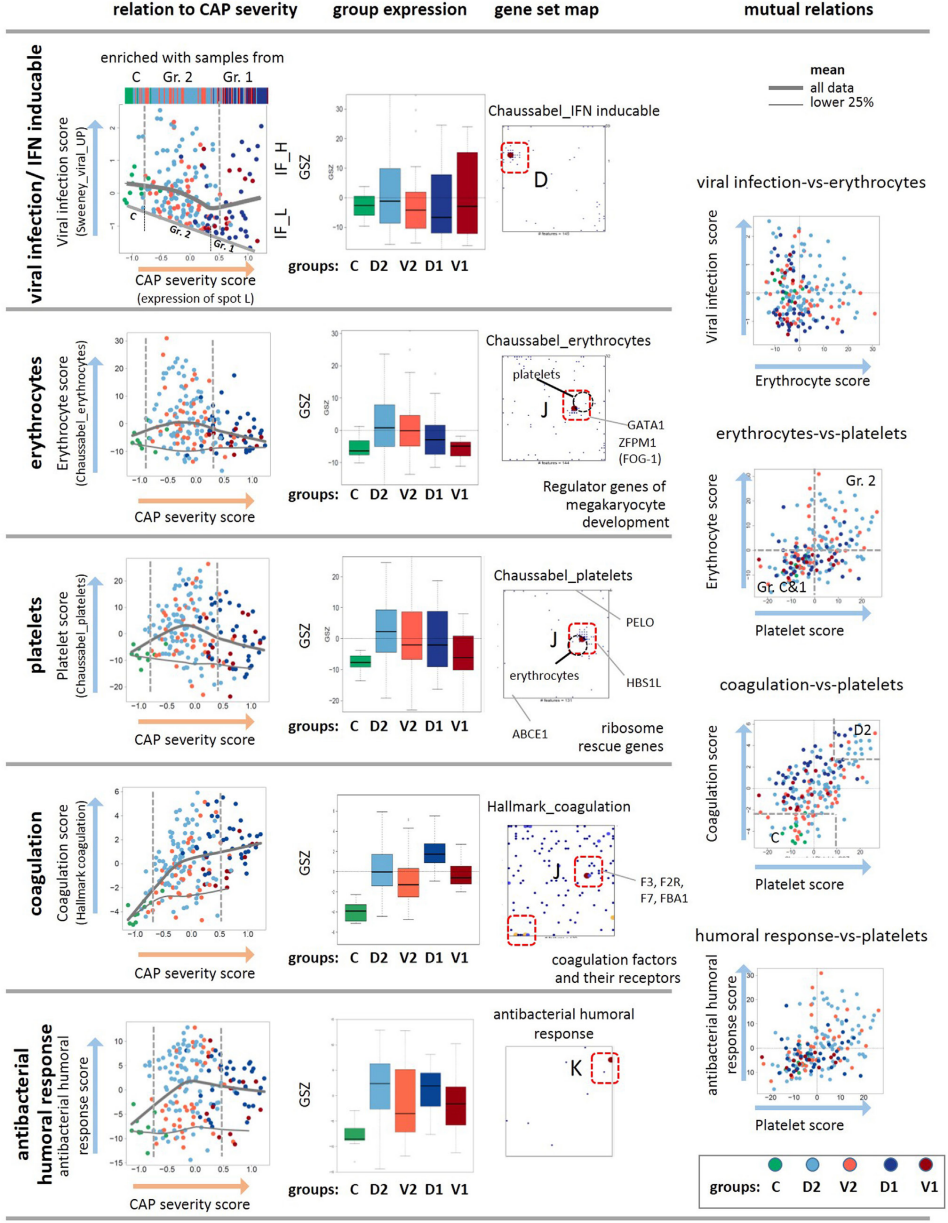
Analysis of the viral, erythrocyte, and platelet signatures as a function of the community-acquired pneumonia (CAP) severity scores. The scores are defined as GSZ-values of selected gene sets as indicated in the figure. The viral, erythrocyte, platelet, coagulation, and humoral response scores show different trends depending on the CAP severity score as indicated by the gray curves. The viral, erythrocyte, and also the coagulation scores positively correlate with each other as indicated by their joint accumulation within the same spot J and the respective scatter plots. The plot of the viral expression score as a function of the CAP severity score reveals a decaying baseline, which suggests exhaustion of interferon induction in more severe CAP cases. The gene set maps also show selected genes that regulate development of platelets and erythrocytes from their common precursors (58), as well as ribosome rescue in both types of cells (59). As a rationale that possibly explains co-expression of mRNA attributed to erythrocytes and platelets. Note also that the positions of the metagenes of highest gene density slightly differ between the platelet and erythrocyte signatures (see the gray circles), which reflects their similar but not identical profiles. The genes of both signatures do not overlap except for one gene (20).

A plot of the expression of the viral signature in Figure 5 (part above) as a function of CAP severity is highly scattered but it also reveals a decaying baseline, which suggests IFN exhaustion after primary viral infection described as host response protecting from secondary bacterial infections (56). Note, that the percentages of bacterial infections in group 1 cases of both cohorts exceed that of the less severe group 2, while that of the viral infections show the opposite trend (9), or in other words, the incidence of bacterial infections in CAP increases with severity while that of viral infections decreases. On the other hand, the mean group-averaged value of the viral score only slightly decreases with CAP severity, but its variance markedly grows, thus suggesting an increasing heterogeneity of the cases regarding their IFN-response (see boxplot in Figure 5). Note also that the CAP groups clearly separate between control, group 2 and 1 along the baseline, while they considerably mix with increasing viral signature, which suggests that the IFN-response contributes to noisiness along the CAP severity axis. Furthermore, the viral expression score and the expression of spot D related to interferon response of D2-group cases exceeds that of V2-cases (Figures 2 and 5), which corresponds to higher percentage of viral infections in D2 compared with V2 [11% in D2 versus 4% in V2, see Table 2 in Ref. (9)]. Hence, the expression differences between both cohorts can partly be associated with the increased percentage of viral and bacterial infections in the discovery cohort [18 versus 12% of Gram positive bacteria and 9 versus 3% of viral infections; see Table 1 in Ref. (9)]. Note that the percentage of viral infections in CAP tends to be underestimated with presumably stronger consequences as suggested by the relative low percentages given above (57).

Spot F accumulating G-protein coupled receptors (*GPCR*) also shows asymmetrical upregulation in samples of the D-cohort *(*Figure 2). *GPCR*s play a vital role in immune regulation (60, 61), e.g., for Treg-cell development and function (62) and T cell receptor and immunoglobulin E signaling (60). Interestingly, cAMP signaling clusters together with *GPCR* suggesting associated functions (Figure 2A) as supported by the fact that cAMP act as core molecules in *GPCR* signaling (63). In summary, the CAP transcriptome expresses footprints of viral and bacterial infections. The former ones form an axis of variance, which is largely independent of the major CAP severity axis while the latter ones concertedly gain with CAP severity. *GPCR* functions seem to be related to these host responses, which, however, requires further studies. In summary, we find close correspondence between IFN-response and viral infection signatures, which show asymmetrical activation in both cohorts considered. Signatures of bacterial infections and ET agree largely with the signature of severe CAP cases.

### Erythroid Cells, Platelets, and Coagulation

Erythrocytes and platelets, although without nucleus, contain a few thousand different mRNAs that are translated in circulating blood and fulfill a broad and multifaceted functional repertoire as inflammatory effector cells (64–66). SOM analysis collects transcripts assigned to both cell types together into one spot J, although the gene sets considered do not overlap (20) (Figure 2). The data show increased expression levels in group 2 compared with the healthy reference state, and decreased levels in group 1 with higher CAP severity (Figure 5), which qualitatively agrees with clinical data on CAP-induced anemia: patients with CAP often exhibit impaired hemoglobin content during acute systemic inflammation and declined prognosis due to disturbances in iron homeostasis (67–69). Interestingly, the gene encoding the hypoxia-inducible transcription factors HIF1A locates also near spot J, thus suggesting co-regulation of hypoxia-related pathways. There is also a significant relation between both thrombocytopenia and thrombocytosis and mortality among the patients with CAP, where the platelet count is considered a better positive than a negative predictor value to the outcome (70). In our data, reduced amounts of mRNA from platelets indeed increases in parallel with the CAP severity score, especially of group 1 (Figure 5). Hence, the expression levels of mRNA from both types of cells seem to correlate with the platelet count and hemoglobin content, respectively. Interestingly, the signature genes of antibacterial humoral response accumulate in spot K showing a similar profile as that of the platelet mRNA with a markedly elevated expression level already in less severe CAP of group 2 (Figure 5). It presumably reflects the role of platelets as inflammatory effector cells in initiating and accelerating vascular inflammatory conditions in pulmonary inflammation (64, 71).

The co-expression of mRNA of platelets and erythrocytes suggest common regulation mechanisms. Note that both cells are derived from a common precursor, the megakaryocyte–erythroid progenitor (58), which in turn is regulated by the hematopoietic transcription factor *GATA1* and its co-factor *FOG1* (72, 73). Both genes are also located in spot J, which indicates their co-expression with the platelet and erythrocyte signature genes (Figure 5). Deficiencies of *GATA1* can cause thrombocytopenia and anemia (74, 75). Another common regulatory mechanism in both cell types associates with ribosome recycling and the recycling factor *ABCE1* decaying in expression with CAP severity, as indicated by its location near spot A (Figure 5). For compensation, the cells need increased levels of the ribosome rescue factors *PELO* and *HBS1L* to support protein synthesis, which is indeed observed as indicated by the location of these genes near the spots L and J, respectively (Figure 5). Hence, common mechanisms during hematopoiesis and ribosome rescue rationalize the co-expression of platelet and erythrocyte mRNA in the CAP transcriptome.

Disordered coagulation represents a third option of concerted expression of mRNA from erythrocytes and thrombocytes because the red blood cells contribute to thrombosis pathophysiology (76). Disordered coagulation is known to accompany severe pneumonia, and it is positively correlated with severity and mortality owing to hemostatic abnormalities (77, 78). The transcriptomic coagulation score (27) indeed shows elevated levels in CAP samples compared with the controls (Figure 5). Hence, expression of mRNA originating from platelets and erythroid cells initially gains in CAP but it reduces for severe CAP cases while the transcriptomic coagulation score seems to further gain, on the average. The used scores offer the options to use them as transcriptional measures of the respective blood characteristics.

### Previous CAP and Sepsis Signatures

**Figure 6:**
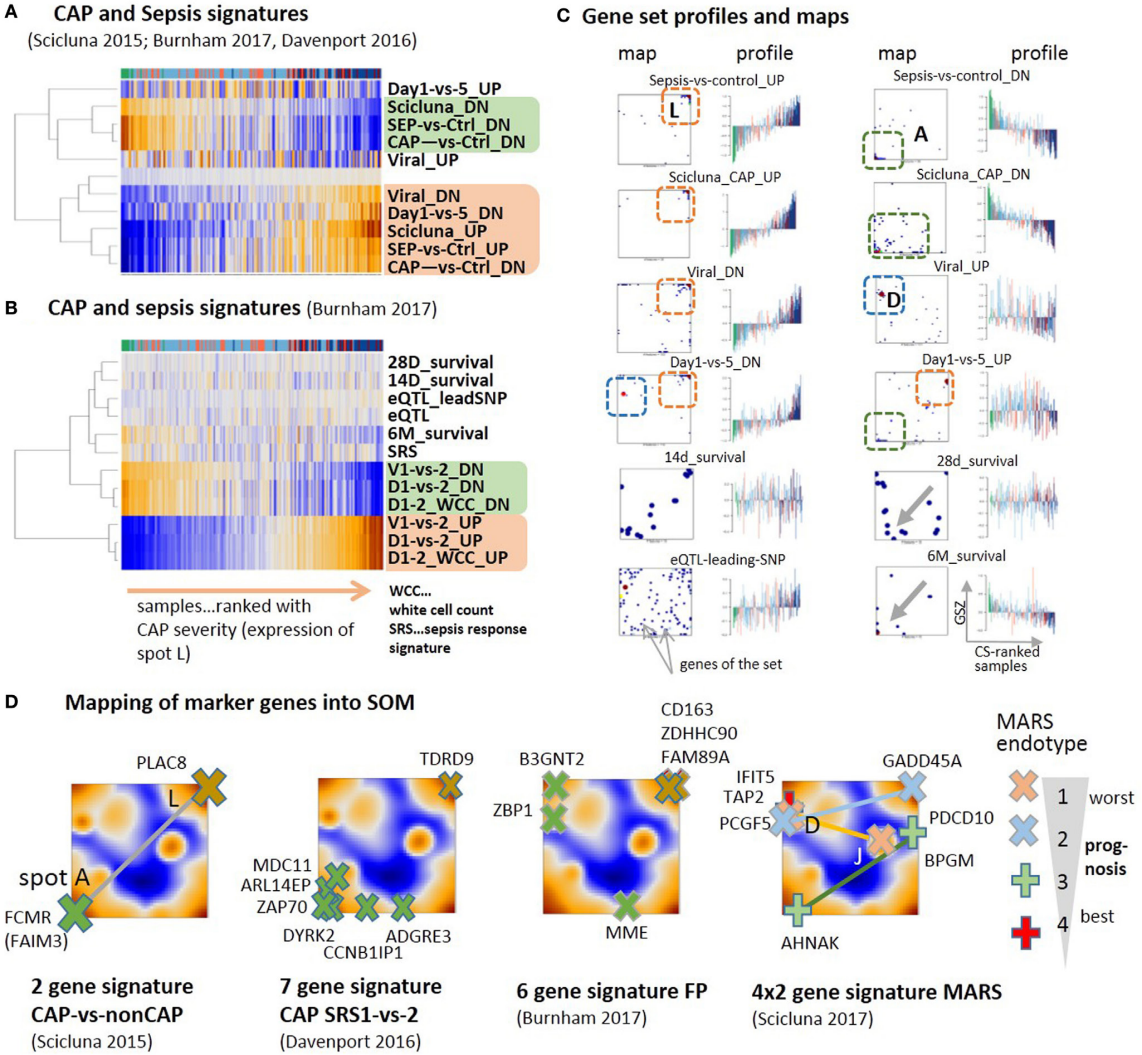
Previous community-acquired pneumonia (CAP) and sepsis signatures were taken from Ref. (9–11, 79) and mapped onto the data analyzed here. **(A,B)** Expression heatmaps (GSZ-score) of the gene signatures mostly show two types of signatures, which antagonistically switch between normal conditions and CAP. **(C)** Profiles and gene set maps (genes are indicated by dots in the maps) reveal further details about the disease course and the noisiness of gene expression. Regions of increased local densities of signature genes were indicated by red and green frames. **(D)** Maps of selected sets of marker genes accumulate in regions of the spots detected here. MARS genes were provided pair-wisely for up- and downregulation in each of the MARS endo-types (11). The pairs are connected by lines as a guide for the eye.

Different sets of signature genes were previously published to serve as markers, e.g., to distinguish CAP from non-CAP cases (79), more severe CAP group 1 from group 2 (9), and to differentiate sepsis due to CAP from FP and also to evaluate the time course of the disease (10). All the signatures estimating the severity of CAP and sepsis show very similar profiles closely resembling that of spot A and L for down- and upregulation, respectively, where the marker genes strongly accumulate in the respective spots (Figures 6A–C). Signatures derived from the time-course of the disease in Ref. (10) (differential expression was counted with respect to the onset of disease) also change along the major dimension of CAP severity indicating an increase of inflammation in the first 5 days after admission to the intensity care unit (“Day 1-versus-5_DN”) and the recovery of normal immune function in 6 months-survivors (“6M_survival”). Survival after 28 and 14 days associates with less clear profiles presumably because cases with improved and worsened courses of the disease mix in this intermediate time interval. Genes activated in CAP upon viral infection and during the 5 days CAP-dynamics accumulate in spot D (Figure 6D), which suggests the influence of confounding factors related, e.g., to the type of infection. The alteration of expression of eQTL genes is compatible with the major dimension of severity due to the susceptibility of these genes for CAP.

A two-gene classifier of CAP versus non-CAP (79) and a seven-gene signature to differentiate between CAP cases of group 1 and group 2 (9) well fit into the spot A versus spot L antagonism defining the main axis of CAP severity (Figure 6D). A signature differentiating CAP from FP (10) and a set of four gene-pairs distinguishing four molecular endo-types of sepsis, called MARS 1–4 (11) partly deviate from this axis and locate in or near other spots, first of all, spot D, related to interferon induction, but also spots E and J. This result suggests stronger association of different processes with the MARS-endo-types and with FP in contrast to CAP. One can interpret the endo-types based on the spot assignments and the location of the respective markers: the endo-type 3 seems to reflect changes along the immune-suppression dimension, the endo-type 2 carries characteristics of viral infections and IFN response on one hand and inflammation and ET on the other. The endo-type 1 with worst prognosis also associates with IFN-response, which, however, antagonizes with alterations of the platelet and erythrocyte mRNA-counts. In turn, the CAP transcriptome thus shows clear characteristics of other forms of sepsis such as FP and the MARS endo-types, however, with a reduced contribution. Taken together, previous CAP and sepsis signatures well agree with the CAP severity dimension defined here by the expression of spot modules A and L. Confounding factors such as the type of infection and the abundance of platelets and red blood cells produce additional heterogeneity with different impact in different stages and types of sepsis. Mapping of previous CAP and sepsis signatures into the CAP transcriptome landscape reveals correspondence between the studies and forms of sepsis regarding the processes involved but also show differences regarding their particular effect-size.

### Reprogramming of Chromatin States

**Figure 7:**
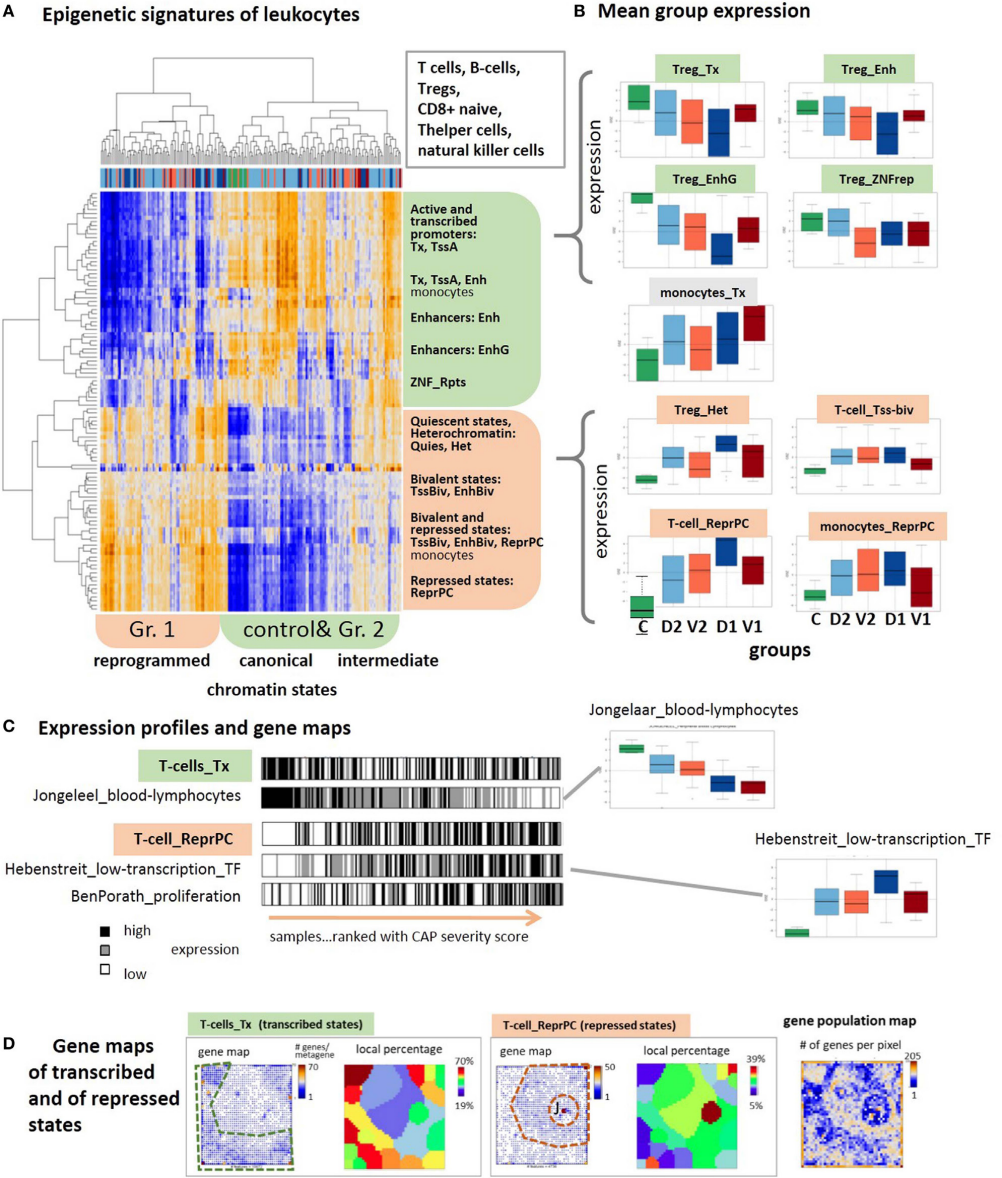
Expression signatures of genes referring to different chromatin states in lymphocytes are evaluated in the community-acquired pneumonia (CAP) blood transcriptome. Chromatin states as defined in Ref. (81) were taken from Ref. (28). **(A)** Two-way hierarchical clustering of the GSZ-heatmap reveals two major groups of chromatin states showing either high expression in predominantly control and group 2 samples (green) or in predominantly group 1 samples (red). Genes upregulated in the former cluster are commonly in transcriptional active states, whereas poised and repressed states on the average are at low expression level. This relation reverses in the latter cluster. **(B)** Boxplots of mean group expression of selected chromatin states show either decaying or increasing expression with increasing severity of CAP. Note the relatively large height of the boxes due to large variability of gene expression (GE) in most of the groups. **(C)** Bar plots of samples ranked with CAP severity underline the increasing and decreasing trends but also the noisy character of GE. Gene sets were taken from Ref. (31, 32, 82). **(D)** Gene maps indicate the accumulation of genes from transcribed and repressed chromatin states in T-cells in different areas of the map, as indicated by the dashed frames. The genes were either shown as dots, or as local percentages in areas determined by *K*-means clustering (12) (number of genes attributed to the state/all genes in the respective area). The population map visualizes the number of genes in each pixel. Chromatin states were defined as follows: active promoters (TssA), transcribed genes (Tx), active enhancer and enhancer-like states (Enh and EnhG), zinc finger proteins (ZNF_rpts), quiescent (Quies), heterochromatin (Het), poised promoters, and enhancers (TssBiv, EnhBiv), repressed polycomb states (ReprPC) (81).

The drastic changes observed in the expression of genes involved in the immune responses suggest the intervention of epigenetic reprograming to condition the response of the immune system. We analyzed sets of genes assigned to distinct chromatin states in a series of different types of healthy blood cells taken from Ref. (28) and discovered systematic changes of their expression levels in the CAP data as possible indications for alterations of chromatin organization between transcribed and silent states [see Ref. (80) for a detailed description of the chromatin states]. The expression data split into two major clusters (Figure 7A): one mostly contains transcribed genes with active promoters, while the other accumulates genes in repressed and poised states. Also, the samples are divided into two major clusters, where one is enriched with controls and less severe cases and the second one with more severe CAP.

Importantly, the expression in the former cluster of samples is in correspondence with the “canonical” assignment of chromatin states in healthy lymphocytes, namely high expression of active genes and low expression of repressed and poised genes. This situation completely reverses in the second cluster of more severe CAP cases, namely nominally transcribed states markedly decay in expression while nominally repressed and poised states become strongly activated. This switching of transcriptional activity suggests changes of the underlying chromatin states, more specifically, activation of repressed and bivalent ones and possibly also deactivation of active ones. Notably, part of the samples form a sort of transition group with intermediate expression levels between the two clusters, which suggests a continuous character of chromatin re-modeling. These antagonistic trends become also evident in the class averaged expression data shown as boxplots in Figure 7B. They are very similar for most of the cell types considered, including T- and B-cells, T-regulatory and T-helper cells and natural killer cells with exception of monocytes whose genes in active and repressed states both increase in parallel with CAP severity.

Regulation of GE in active states can be achieved by alternative mechanisms, e.g., *via* “classical” transcription factors, which potentially overlay with the epigenetic mechanisms studied here. The profiles of signature sets of lymphocytes (31) and of transcription factors associated with weakly expressed genes under healthy conditions (82) closely resemble the profiles of active and repressed chromatin states, respectively (Figure 7B, part below). This agreement lets us conclude that the expression characteristics of the chromatin states indeed reflect essential expression characteristics of the blood cells studied. The relative large SD of the group-related expression values reflects a large noisiness of the expression of chromatin states (Figure 7B). The barcode patterns in Figure 7C further illustrates this in terms of scattered black and white stripes referring to high and low expression levels, respectively. Importantly, gene maps show that the expression landscape divides roughly into two major areas that accumulate either active or repressed states referring to low and high severity of CAP, respectively (Figure 7B, part below). In summary, blood transcriptomics reveals footprints of progressive chromatin re-organization with CAP progression, where silent states in healthy blood on average become activated and active states decay in expression.

### Chromatin Modifying Enzymes

**Figure 8:**
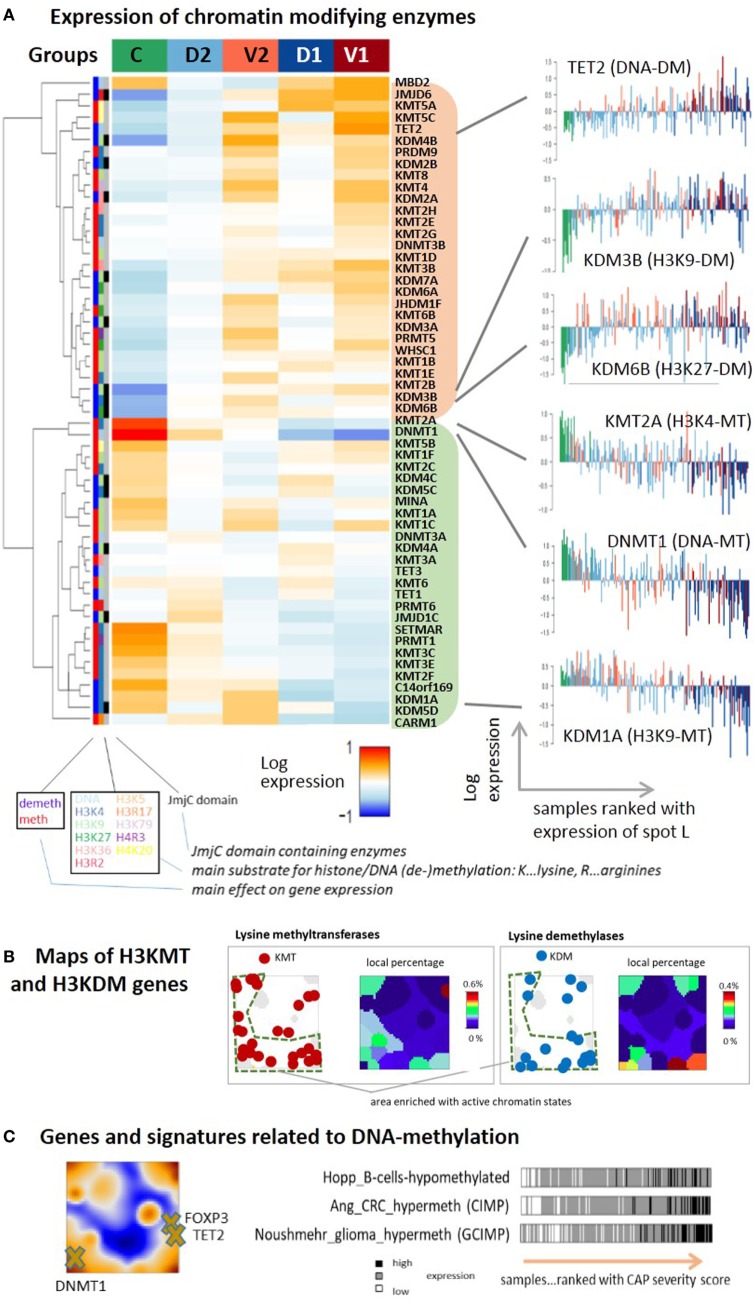
**(A)** Expression heatmap of chromatin modifying enzymes in the five sample groups: the gene expression data of methyltransferases (MTs) and demethylases (DM’s) of DNA cytosines, histone lysine (H3K), and arginine (H3R) side chains split into two main clusters down- or upregulated in the control group compared with the community-acquired pneumonia groups. The plots in the right part show expression profiles for selected enzymes with single sample resolution. **(B)** Maps of genes encoding lysine MTs and demethylases of H3K4, H3K9, H3K26, and H3K36, and of their local percentage among all genes. The genes are accumulated in the region assigned to active chromatin states and are depleted in the region assigned to repressed and poised states as shown in Figure 7D. **(C)** Genes and gene signatures related to DNA methylation suggest activation of repressed and poised genes by DNA-demethylation.

Chromatin organization is regulated by a large battery of chromatin modifying enzymes whose transcriptional activity is expected to vary between the CAP groups in parallel with the changes of chromatin states reported in the previous subsection. We evaluated GE of more than 50 chromatin modifying enzymes either catalyzing methylation [methyltransferases (MTs)] or demethylation (demethylases, DM) of lysine or arginine side chains of the histone-component H3, or directly catalyzing methylation and demethylation of DNA cytosines usually in CpGs. The heatmap in Figure 8A reveals two main clusters of enzymes with either up- or downregulated expression in the control group and group 2 on one hand, and mostly antagonistic expression in group 1 of the more severe CAP cases on the other, i.e., a similar clustering of cases as for the expression of chromatin states discussed above. For example, the expression of the histone demethylase *KDM6B* upregulates with CAP severity. It de-methylates repressive H3K27me3 marks in repressed and poised gene promoters giving rise to transcriptional activation of the affected genes, suggesting a trend that corresponds to the changes of initially silent chromatin states. *KDM6B* was recently shown to link microbial stimulus with epigenetic gene regulation during host responses (83). The MTs *SETMAR, KMT3C* (*SMYD2*), *KMT3E* (*SMYD3*), *KMT2F* (*SETD1A*), all catalyzing methylation of the histone marks H3K4me1-me3 associated with active promoters concertedly decay in expression, thus showing a trend that corresponds to the changes of initially active chromatin states. However, the expression of a series of demethylases of H3K4me1-me3 such as *C14orf169* (*RIOX*), *KDM1A* (*LSD1*), and *KDM5D* (*JARID1D9*), accomplishing the opposite role, also decays with CAP severity, which makes the picture more puzzling. In general, one finds a mix of different enzymes writing or erasing repressive (H3K27me3, H3K9me3) and active (H3K4me3, H3K36me3) marks in both clusters of decreasing and increasing expression with CAP severity. Overall, this result suggests massive deregulation of the machinery of chromatin modifying enzymes in the course of CAP, which parallels chromatin re-organization discussed in the previous subsection [see Ref. (84) for a general review]. Mapping of lysine methylases and de-methylases into the SOM portraits reveals an asymmetrical distribution with a larger density in the region assigned to active chromatin states (Figure 8B, compare with Figure 7D), which suggests that these enzymes were preferentially transcribed from active states under healthy conditions.

Changes of chromatin states due to alteration of enzyme activity in CAP are expected to also modify DNA-methylation. It represents another epigenetic level of transcriptional regulation, where poised and repressed states often tend to become methylated and active states tend to loose DNA methylation marks. DNA methylation is directly governed by DNA (de-)methylating enzymes (85). We find that the expression of the DNA-methyltransferase *DNMT1* ensuring maintenance of DNA methylation after cell division decays with CAP severity while the expression of the DNA dioxygenase *TET2* that de-methylates DNA (86) increases (Figure 8A, right part). *DNMT1* expression was shown to correlate with pro-inflammatory cytokine production in the peripheral blood monocytes (87) and the release of LPS-induced pro-inflammatory cytokines, such as *TNF-*α and *IL-6* in macrophages (88). Moreover, de-activation of DNMT1 might lead to M2 polarization (89), as suggested by our data. *TET* genes, particularly *TET2*, play an antagonistic role by restraining inflammatory GE in macrophages (90) and its level was shown to decrease after viral infection (91). The trends observed for *DNMT1* and *TET2* expression suggest loss of DNA-methylation marks with CAP severity. Data on DNA-methylation in pneumonia and, particularly, CAP are rather scarce or even missing to our best knowledge. One study found that lung inflammation indeed leads to DNA hypomethylation in the lung and blood, which suggests disease development (92). Another study reported DNA demethylation of the *FOXP3* gene and other key genes typifying the Treg lineage (92) with potential impact for therapy (93). *FOXP3* co-localizes with *TET2* in our CAP-SOM, indicating co-expression, which is compatible with activation of *FOXP3* by DNA de-methylation (Figure 8C). Interestingly, the expression of signature genes with aberrant methylation patterns in colorectal cancer [CIMP-genes (94)], gliomas [GCIMP-genes (95)], and also B-cells (96) increase with CAP-severity. These traces in the CAP transcriptome indicate that similar mechanisms and gene groups are susceptible for alterations of DNA-methylation in different tissues and diseases, which particularly applies to genes with poised and repressed promoters having impact for cell fate decisions, development, and differentiation (17, 96–98). We hypothesize that genes in repressed and poised states de-methylate with possible consequences for immune response. In summary, we find extensive transcriptional deregulation of chromatin modifying enzymes in the CAP transcriptome presumably affecting chromatin organization and predicting widespread alterations of DNA methylation.

### Deregulation of Pathway Activities

So far, we have mined functional knowledge using gene set analysis that calculates mean expression levels of signature genes but ignores potential interactions between them (23). PSF analysis considers this information in terms of pathway topologies (30). The PSF activity heatmap of KEGG-pathways divides into two major clusters of processes either activated in the controls and deactivated in CAP or vice versa. The B-cell receptor (BCR) and T-cell receptor (TCR) signaling pathways, natural killer cell-mediated cytotoxicity, cell adhesion, *JAK-STAT, RAS, PI3K*-*AKT*, and *p53* signaling pathways belong to the former ones (Figure S7 in Supplementary Material). They reflect deactivation of immune functions. Among the pathways activated in CAP are *MAPK*-, *HIF1*-, toll-like receptor-, *NFkappaB*-signaling pathways, olfactory transduction, glycogenesis and gluconeogenesis, glucosaminoglycan degradation, water adsorption, and drug metabolism.

The detailed evaluation of selected pathways reveals a more diverse situation (Figures S8 to S17 in the Supplementary Material). Part of these pathways, namely Fc-gamma-mediated phagocytosis and GnRH signaling, become strongly activated in CAP in all of their branches (Figures S9 and S10 in the Supplementary Material). These pathways are among the top deregulated pathways in a meta-analysis of a series autoimmunity and autoinflammation diseases (99). Conversely, most of the alterations in other pathways refer only to certain branches. For example, *HIF1*-pathway leads to activation of *CREBBP* and *ARNT* that then lead to expression of several genes leading to diverse functional outcomes, such as *VEGF*-signaling and adaptive response to hypoxia (Figure S15 in Supplementary Material). On the other hand, *NFkappaB*- (and/or *MAPK*) is represented as a branch in multiple pathways such as the TCR-, BCR-, toll-like receptor-, and cytosolic DNA-sensing-signaling pathways. These processes either become activated or deactivated in CAP depending on the pathway they are involved in (e.g., deactivated in TCR signaling, Figure S8 in Supplementary Material or activated in toll-like receptor signaling, Figure S11 in Supplementary Material, partly also BCR-signaling, Figure S13 in Supplementary Material), owing to different entry points of the signals (e.g., *MYD88* in toll-like receptor signaling), due to involvement of diverse regulatory processes and also due to different requirements to control transcription and cell survival under stress. Hence, activation of the pathway branches is dependent on the particular context.

Note that the sets of signature genes discussed in the previous subchapters often accumulate within or near one spot because they are usually selected by applying the criterion of “concerted up- or downregulation.” In contrast, the pathways consider activating and repressive interactions between the genes that give rise to correlated, anti-correlated, and even more complex mutual expression changes between member genes (100). As a consequence, the maps of the pathway genes often accumulate in more than one spot-area, as a rule of thumb (e.g., see Figures S13 and S16 in Supplementary Material). Especially, spots H and I in the right lower corner of the map are occupied by genes of the BCR-, HIF1-, NFkappaB-, and MAPK21-pathways, which attributes these spots to activation pattern of these pathways (Figures S13, S15, and S17 in Supplementary Material).

Taken together, pathway analysis estimates activation of genes in the context of their wirings, e.g., in signaling cascades and thus it complements gene set analysis. The pathways considered clearly divide into two clusters with antagonistic alterations of their mean activity in CAP.

### SOM Portrayal Suggests a Novel Stratification Scheme With Potential Prognostic Impact

**Figure 9:**
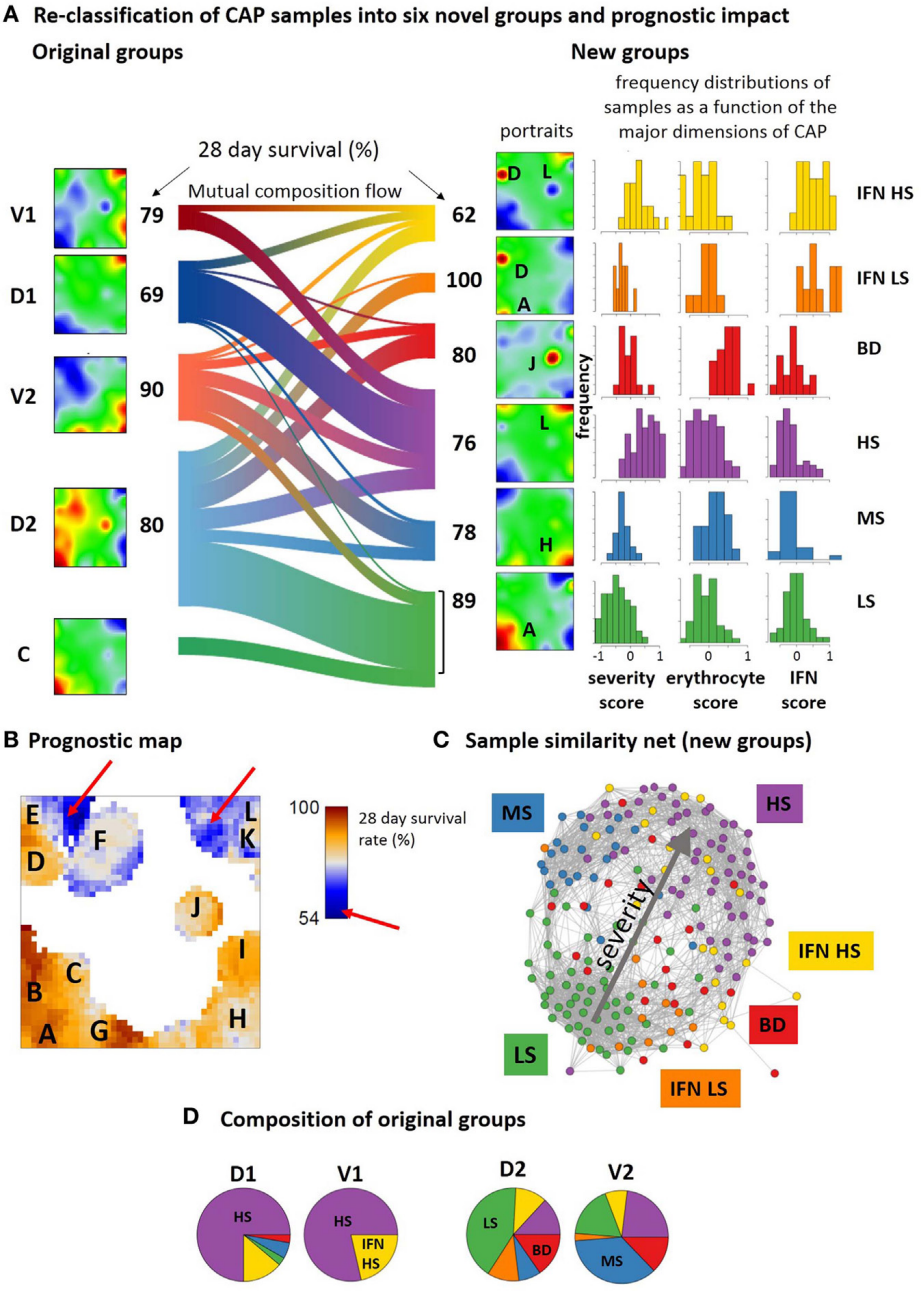
Re-classification of community-acquired pneumonia samples into six novel groups and their prognostic impact. **(A)** The sankey diagram (R-program “riverplot”) illustrates the composition flow between the original and novel classes [low, medium and high severity, LS, MS, HS, respectively; blood disturbances (BDs); IFN high and low or middle severity, IFN LS and IFN HS, respectively], while the class-averaged mean portraits visualize the respective expression landscapes. The new subtypes differ with regard to severity levels (low, medium, high, spots A, H, and L) and the expression of spots J and D being assigned to BDs and interferon response, respectively (see also Table S4 in Supplementary Material). **(B)** The prognostic map links the expression level with the 28-day survival rate where the latter is calculated as the percentage of patients expressing the respective metagene and surviving 28-days after admission in intensive care unit. **(C)** Re-coloring of the correlation net according to new classification illustrates the asymmetrical distribution of the novel groups with respect to the discovery and verification cohorts (compare with the similarity net in Figure 1A). **(D)** The pie charts show the composition of the original groups with respect to the new ones. Note that about 65% of group 1 cases are collected in the novel HS group, while the remaining cases are mainly assigned to IFN HS. In contrast, about 50% of group 2 cases consist of LS and MS cases, while the remaining half of the cases distribute over the remaining groups.

The SOM analysis of GE modules in CAP-induced sepsis indicates considerable heterogeneity of responses that cannot be fully explained by division into two groups of low and high immune-suppression (9, 10). This classification considers the severity dimension of sepsis but not explicitly the IFN-response dimension related to viral infections (spot D) and the expression of mRNA originating from erythrocytes and platelets (spot J). Based on the spot expression profiles and subsequent class discovery, we re-classified the CAP cases into six novel groups (Figure S18 in Supplementary Material). The mean portraits of the new groups (Figure 9A, see also Figure S2B in the Supplementary Material for the sample portraits) reveal specific spot patterns that indicate the most elevated transcriptional modules in each of the novel groups, which were assigned to low and high severity (spots A and L, respectively), interferon response (spot D), blood disturbances (BD) (spot J), and medium severity (spot H). The frequency distributions of the CAP severity score show pronounced differences between the groups that combine with specific differences in the distributions of the erythrocyte and viral infection scores, thus providing a stratification scheme along three dimensions of the CAP transcriptome. The novel groups distinguish CAP cases of low (LS), middle (MS), and high (HS) severity levels, CAP cases showing high expression of the BD spot J and mostly medium levels of severity, and CAP cases with strongly expressed interferon response that further divide into a group of low (to middle) and high severity score (IFN LS and IFN HS, respectively). The LS group accumulates slightly younger patients (mean age 59 years, *p* < 0.05, Fishers exact test, Table S3 in Supplementary Material) while IFN-LS collects slightly elderly ones (71 years, *p* < 0.1). The 28-day survival rate is best for LS (89%, *p* = 0.07, Fishers exact test) and worse for HS (76%) and MS (78%) groups meaning that cases with intermediate values of the severity score have still relatively bad prognosis. Interestingly, combination of IFN response with high levels of immune-suppression in IFN HS results in worst prognosis (62%, *p* = 0.02) while all patients in IFN LS survived (100%, *p* = 0.09). Please note also that the MS, BD, and IFN-LS groups show very similar frequency distributions of the severity score while their expression spot patterns and survival rates markedly differ. This result illustrates the potential prognostic impact of the additional dimensions of CAP. For validation of these results in terms of the two-cohort concept applied in Ref. (9, 10), we further stratified the 28-day survival rates of the new groups with respect to samples taken from the discovery and validation cohorts (Table S4 in Supplementary Material). Both cohorts show worst prognosis for IFN HS and best prognosis for IFN LS and LS groups. The combination of enhanced IFN response and of strong immune-suppression as in IFN HS and an associated poor prognosis was also observed in FP and the MARS endo-types 3 and 4 (Figure 6D). This result further supports the prognostic impact of the new groups namely that IFN response due to viral infections worsens prognosis in severe forms of sepsis.

The prognostic map in Figure 9B visualizes the 28-day survival rate with pixel-resolution. It shows that some of the spots associate with poor outcome while others associate with good prognosis. For example, patients referring to samples that overexpress spot A, B, or G were almost all alive after 28 days while activated genes near spot L associate with a survival rate of about 50% only. Accordingly, the 28-day survival rate changes nearly by a factor of two in dependence on the region of the map. Note that areas of worst prognosis (see red arrows in Figure 9B) are slightly shifted compared with the spot regions of maximum variation of GE (compare Figure 9B and Figure 1B). In other words, genes with largest prognostic impact do not necessarily show largest effect of differential expression between the groups.

The composition flow diagram in Figure 9A illustrates that original and new groups largely mix where, however, cases of group 1 and group 2 accumulate cases of the novel HS and LS groups, respectively (Figures 9C,D) and cases of the discovery cohort accumulate in the two IFN-groups. This again illustrates the asymmetry of the two cohorts with respect to their IFN-response. Taken together, stratification of CAP cases according to the level of severity, IFN-response, and BDs results in a novel stratification scheme with prognostic impact. Prognosis is further specified using a novel type of prognostic map that links the transcriptome landscape with clinical information.

### Transcriptional Dynamics

Next, we analyzed the transcriptome dynamics of CAP patients who sequentially provided blood at days 1, 3, and 5 after admission into ICU. Individual SOM portraits of selected patients indicate typically the improvement of their health status in terms of the continuous decay of the severity score during these 5 days while the erythrocyte score seems to increase (Figure S20A in Supplementary Material). The mean SOM portraits for each time point support this observation: at day 1, spots L and D that are indicative for high severity and IFN-response, respectively, are highly expressed while during days 3 and 5, the expression shifts toward spots A (immune response) and J (erythrocytes and platelets), respectively (Figure S20B in Supplementary Material). Trivially, these patterns agree with the signature genes for days 1 and 5 extracted in Ref. (10) accumulating in the respective areas of the SOM (see the gene maps “day 1-versus-5_UP” and “day 1-versus-5_DN” in Figure 6). The composition flow diagram in Figure S20C in Supplementary Material confirms these changes, namely, that the amount of samples in the low severity groups LS and IFN-LS gains at day 5 compared with days 3 and 1. Interestingly, the BD- and IFN-groups seem to get involved more strongly at day 3 and partly at day 5 that suggests the delayed response to viral infections and of BDs compared with the sepsis score, which shows maximum effect at day 1. The dynamic information can serve only as a first glimpse on the course of the disease in the first days after admission into ICU because the number of patients with three serial blood samples for which temporal information is available is relatively small (*N* = 15).

## Discussion

As for cancers, the concept of using high-throughput omics data to analyze the multidimensional character of the transcriptome landscape of sepsis and to disentangle the molecular heterogeneity of this disease into molecular subtypes is expected to become clinically useful (22). In the first part of the paper, we discovered the data structure by means of the expression portraits of all samples, summarized them into an expression landscape, extracted expression (“spot”-) modules, and analyzed functional signatures where we kept the group labels as taken from the original study (10). Based on these results, we propose a novel stratification scheme of six CAP subtypes of prognostic impact.

Our analysis of the CAP-transcriptome confirmed previous results, namely that immune suppression and ET constitute the major footprints of this disease in the blood transcriptome (9, 10). Consistently with previous findings, differential analysis of the transcriptome provided a set of marker genes distinguishing between cases of low and high severity (SRS groups 2 and 1) that associate with better and worse prognosis for survival, respectively. However, our analysis methodology using self-organizing maps allowed us to significantly widen the interpretation of the pathobiology of CAP and associated sepsis and suggested new transcriptomic footprints related to disease development. Particularly, we found that:

– the epigenetic machinery (DNA-methylation and histone modification) plays an important role in the pathophysiology and correlates with activation of transcriptomic modules defining CAP severity and survival;
– interferon response and mRNA originating from erythrocytes and platelets constitute two additional major dimensions of variations of the blood transcriptome closely related to disease pathogenesis;
– these additional dimensions relate, at least partly, to viral infections and BDs and/or respiratory problems, respectively;
– the additional dimensions were found also in other forms of sepsis what makes them relevant in a more general context (10, 11);
– marker genes taken from previous sepsis and blood-transcriptome studies are applicable to CAP to quantify the major dimensions of this disease.

### Expression Portrayal Disentangles the Multiple Dimensions of the Transcriptome

**Figure 10:**
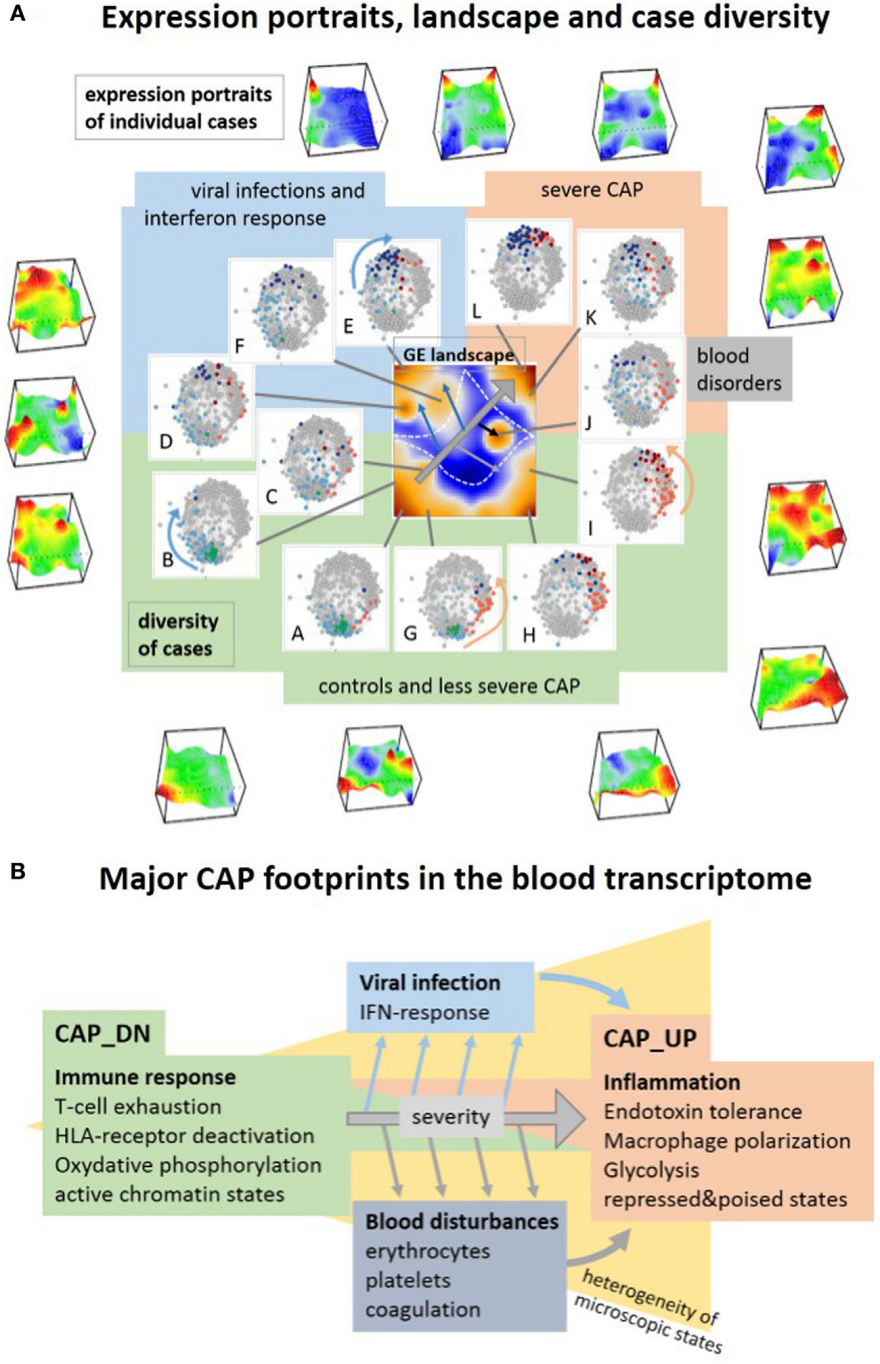
Visualization of the transcriptome landscape on three levels **(A)**, namely “personalized” expression portraits, a “feature” gene expression (GE) landscape and a sample diversity landscape and schematic overview of the major community-acquired pneumonia (CAP) footprints in the blood transcriptome **(B)**. **(A)** The portraits show up- and downregulated modules of correlated genes in red and blue, respectively, where the vertical coordinate visualizes the expression and the horizontal one the self organizing maps-mosaic. The GE “feature” landscape summarizes these spots as maroon areas of variant GE (see also Figure 1B). For each spot A–L, we colored the cases showing this particular spot in the diversity network (see also Figure 1A). Spot activation associates with groups of samples in the network: increasing severity of CAP activates spots roughly along the arrow in the GE landscape, which in turn associates with cases located in direction of the arrows shown in the similarity nets.

We here applied the self-organizing map (SOM) portrayal method to characterize the CAP blood transcriptome. It provides information on three major levels, first, in terms of “personalized” transcriptomic portraits of each case, second, in terms of an expression landscape indicating a collection of so-called spot modules, and third, the diversity landscape of cases studied (Figure 10A). For a holistic view on the CAP-expression landscape, we trained all samples of the original study (9, 10) together in one SOM and decomposed overall transcriptomic landscape into one dozen spots A–L of co-regulated genes. Their functional context has been annotated using a collection of gene signatures covering a wide range of functional categories. We also mapped a battery of previously generated signatures related to CAP, sepsis, the type of infection, and different blood cells to disentangle the expression landscape of CAP. This way it enables to assign the functional context of a signature and to judge its heterogeneity. It also enables to “read” the individual portraits and evaluate the contribution of disturbed modules (for example, indicate high or low levels of platelet mRNA, activated or deactivated IFN-response or immune suppression) for each individual case, and thus a personalized combinatorics of different features with potential diagnostic impact (see the portraits in Figure 10A). Thus, the advantage of using SOM machine learning here is that it identifies hidden multidimensional structures in complex transcriptomic data, which can be assigned to the major dimensions of the CAP transcriptome in terms of distinct “spot”-clusters of co-regulated and functionally related genes. Such multidimensional transcriptional response patterns typically cannot be extracted with sufficient resolution using supervised comparisons, e.g., between CAP and healthy controls. The multidimensional expression landscape transforms into a series of substrata of CAP cases according to the activated spots as indicated in diversity maps in Figure 10A. Mapping of marker signatures for different forms and endo-types of sepsis into the CAP transcriptional landscape reveals correspondence with the three major dimensions identified here where they, however, can combine with different weights. Reductionist sets of (only a few) marker genes, e.g., for CAP and FP, are usually restricted to one of the dimensions, which makes them prone to overlook other important dimensions of the disease. The prognostic map provides a novel option of linking the transcriptomic landscape with clinical data with potential impact for marker selection. In summary, SOM analysis disentangles the multidimensionality of the CAP transcriptome and extracts modules of co-regulated genes with potential impact for the selection of candidates for molecular markers.

### Three Major Dimensions of CAP in the Blood Transcriptome

Transcriptional profiling of peripheral blood reflects physiological and pathological events occurring in the course of sepsis framed within CAP. It allowed us to identify footprints of immune suppression, of specific host responses to infections, of BDs, and of epigenetic effects. Immune suppression constitutes the main dimension of transcriptional alterations in the CAP blood transcriptome. It associates with T-cell exhaustion and HLA and chemokine receptor deactivation paralleled by ET and macrophage polarization, activation of CD4 cells and of metabolic conversion from oxphos to glycolysis (Figure 10B). Elevated glycolysis is the metabolic basis for trained immunity, providing the energy and metabolic substrates for the increased activation of immune cells in analogy with the Warburg effect in cancer (34, 101). The major expression footprint reflects rather continuous alterations of microscopic states between the healthy controls and severe CAP cases than a stepwise change between healthy, moderate, and severe CAP. This major dimension of CAP severity associates with sepsis and a bad prognosis (9). Dynamic measurements further show that sepsis severity decays on the average during the first 5 days in ICU. The major severity dimension is modulated by two additional characteristics constituting a second and third dimension of variations of the CAP blood transcriptome. The first one is the IFN-response dimension partly related to hosts response to viruses. It is highly variable among the cases and suggests IFN exhaustion along the major severity axis. The second characteristic dimension reflects the level of mRNA originating from erythrocytes and platelets. It suggests disturbances in blood cellular profiles such as enhanced and reduced levels of mRNA from both cell types in less and more severe CAP, respectively, and also the increase of coagulation signatures suggesting coagulopathy. The association of increased expression within these modules and less severe CAP possibly reflects their delayed response at days 3 and 5 after admission into ICU due to viral infections and/or respiratory comorbidities and oxygen requirements.

We found systematic differences between the discovery and validation cohorts regarding the level of IFN-response (especially in low-severity group 2 samples) that is higher in the discovery cohort and associates with a higher percentage of viral infections. The division of samples into these strata thus enables validation of the major severity-dimension but not of the second dimension related to IFN-response. In summary, our analysis confirms previous results (9–11) regarding the major dimension of transcriptional changes in CAP severity, but it also identifies two additional, partly independent dimensions related to IFN response and BDs.

### The Impact of Epigenetics

The blood transcriptome of CAP reveals footprints of chromatin re-modeling compared with the healthy state where silent chromatin becomes transcriptionally activated and transcribed chromatin becomes repressed. Extensive transcriptional deregulation of chromatin modifying enzymes seems to act as one of the driving forces of chromatin re-organization. It is known that chromosomal reprogramming *via* histone (de-)methylation indeed parallels immune response and re-structures cellular programs and plasticity, for example, to induce ET (34) and to train monocytes for immune response (102, 103). Hence, our results support the view that epigenetic effects constitute an important mechanism of genomic regulation in the course of sepsis, e.g., to transiently sensitize immune response and subsequently to suppress immune reactions (34). This result supports the recent finding that expression-associated single nucleotide polymorphisms accumulate in regulatory elements of endotoxin tolerant genes and in histone marks for active promoters (9). Moreover, changes in DNA methylation are known to play a critical role in the division of hematopoietic stem cells into the myeloid and lymphoid lineages and in the establishment of specific functionalities in terminally differentiated cell types (104). Based on our results, we predict extensive changes of DNA methylation in the course of sepsis with potential impact for marker selection and functional characterization, which presumably will complement and extend the information extracted from the footprints in the transcriptome. Our study thus adds indications for an important role of epigenetic mechanisms in CAP with possible consequences for diagnostics and treatment (104).

### Novel Stratification of CAP Based on Functional Dimensions

The SOM-analysis of the CAP expression landscape suggests a modified stratification scheme that in addition to high and low levels of sepsis severity takes into account the level of interferon response, high levels of mRNA originating from erythrocytes and platelets, and also a class of medium sepsis severity. The six novel classes diversify the potential prognostic options as, for example, combination of IFN-response with high sepsis severity seems to worsen the 28-day survival prognosis. The novel stratification also opens options for dynamic studies of the course of the disease. These data available suggest different dynamics of the responses of the major dimensions. Larger cohorts and more extended dynamic measurements are, however, required to verify these results. Overall, our analysis enhances the resolution of transcriptome footprints of CAP and it provides opportunities for selecting sets of transcriptomic markers that might be of interest for clinical applications.

## Author Contributions

Conceived the study, performed analyses, and wrote the manuscript: LH and HB; interpretation and methods development: LH, HL-W, LN, AA, and HB. All authors contributed to the final version of the manuscript.

## Conflict of Interest Statement

The authors declare that the research was conducted in the absence of any commercial or financial relationships that could be construed as a potential conflict of interest.
