# Supplementary material for: Footprints of Sepsis Framed Within Community Acquired Pneumonia in the Blood Transcriptome
Source: Front Immunol. 2018 Jul 17;9:1620. doi: 10.3389/fimmu.2018.01620 (PMC6056630; doi:10.3389/fimmu.2018.01620)
Supplement: Supplementary file 3 [file Data_Sheet_1.docx]

Supplementary Figures and Tables

Footprints of Sepsis framed within Community Acquired Pneumonia in the blood transcriptome

Lydia Hopp ^1^*, Henry Loeffler-Wirth ^1^, Lilit Nersisyan ^2^, Arsen Arakelyan ^2^, Hans Binder ^1^

1. Interdisciplinary Centre for Bioinformatics, Universität Leipzig, Härtelstr. 16 – 18, 04107 Leipzig, Germany; E-Mails: hopp@izbi.uni-leipzig.de (L.H.); binder@izbi.uni-leipzig.de (H.B.); wirth@izbi.uni-leipzig.de (H.W.)
2. Group of Bioinformatics, Institute of Molecular Biology, National Academy of Sciences, Yerevan, Armenia

***** Author to whom correspondence should be addressed


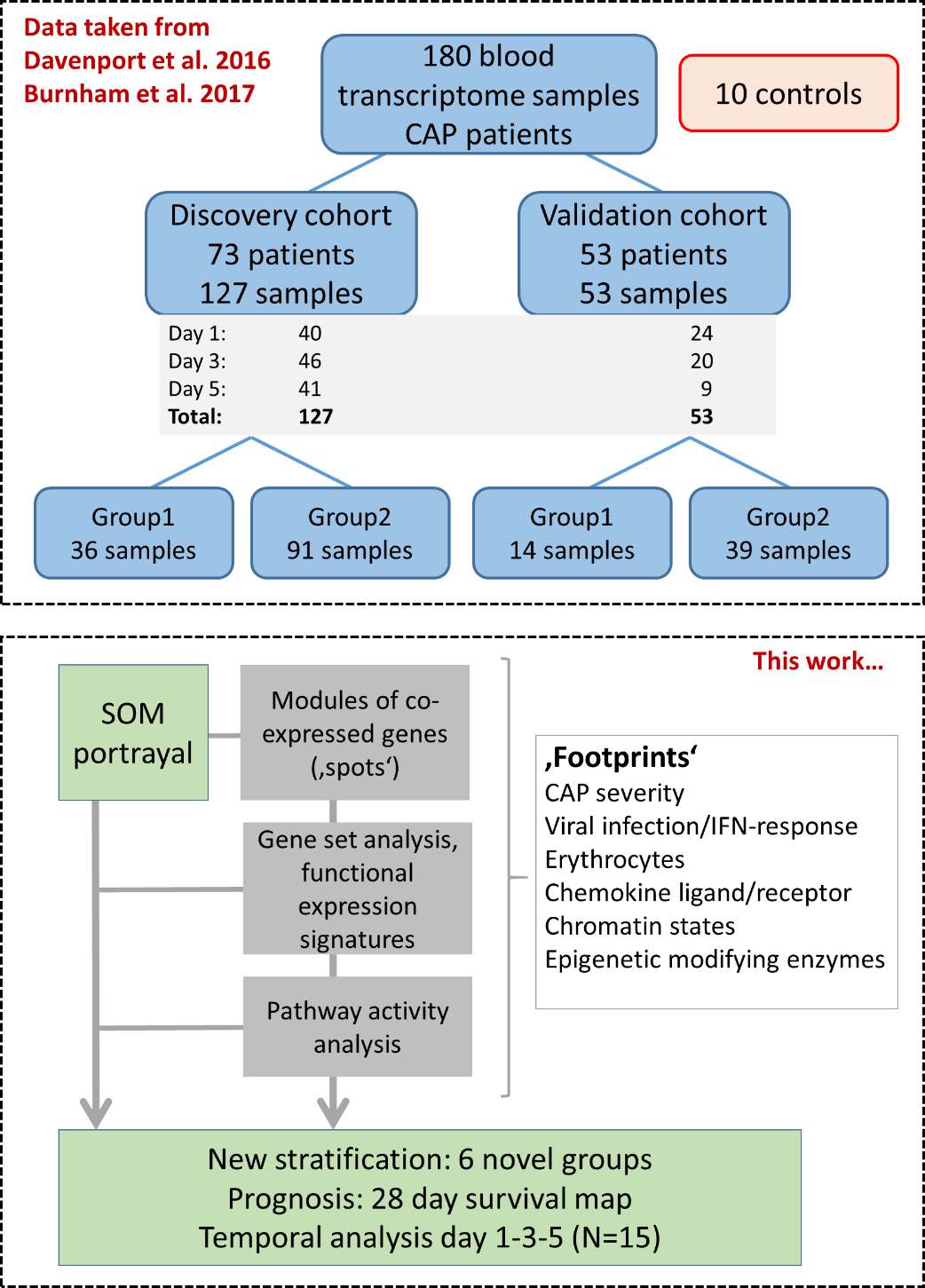


Figure S 1: Schematic of the composition of the cohort data taken from (9, 10) (the scheme is adapted from Figure E1 in (10)) and of the main analysis steps performed in this work. Note that all CAP samples and the controls were analyzed together in one SOM training in order to identify all relevant expression modules (‘spots’) that constitute sets of co-expressed genes identified as described in (12) and implemented in (24). The new stratification of the CAP samples makes use of these expression modules. It takes into account the multidimensional nature of the expression landscape. Preprocessed gene expression data were downloaded from the ArrayExpress database under accession numbers E-MTAB-5273 for the discovery cohort and E-MTAB-5274 for the validation cohort. Preprocessing included background signal subtraction, outlier sample removal, inclusion of probes exceeding a detection rate threshold and normalization using the variance stabilization and normalization method (10).


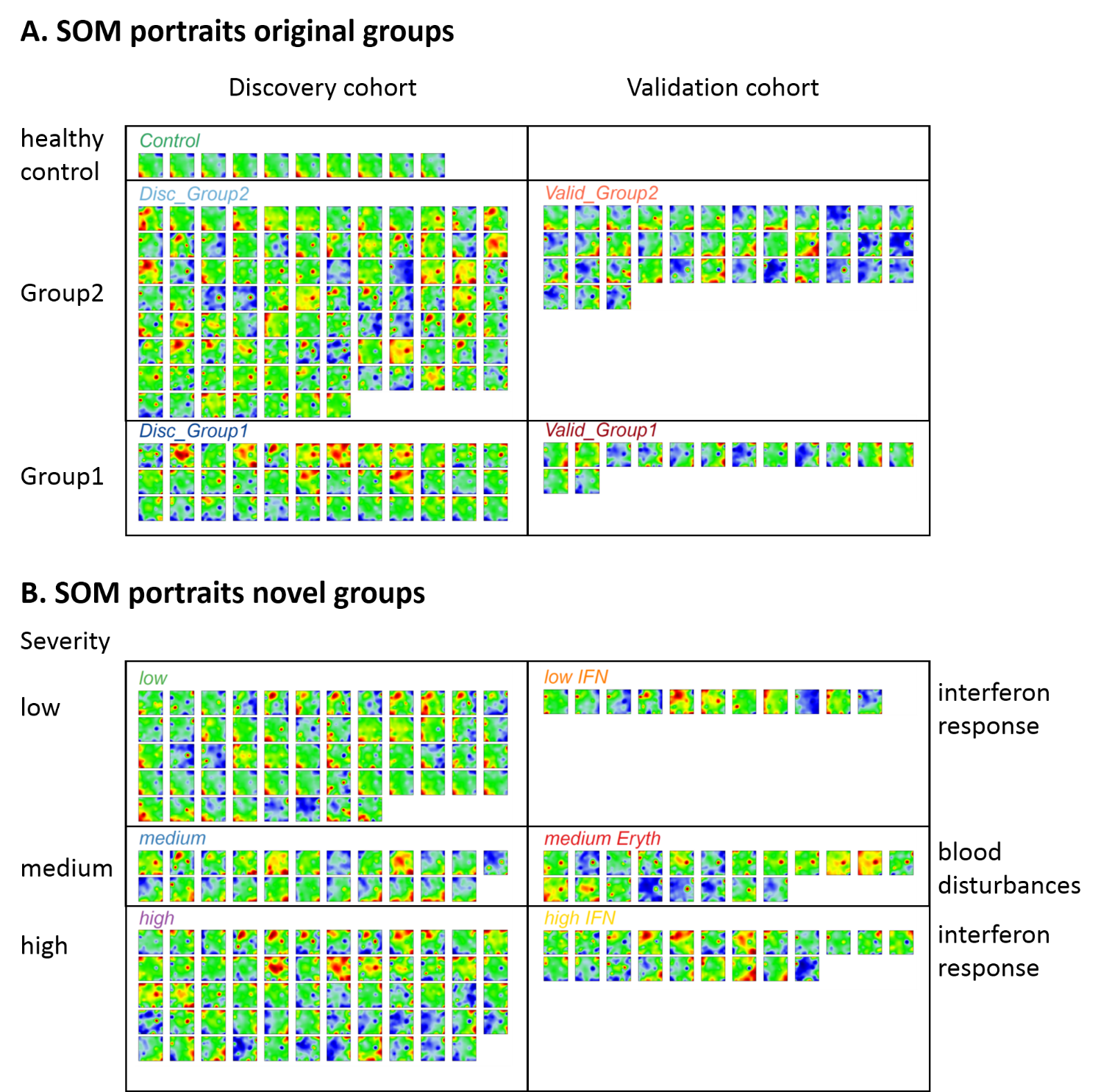


Figure S 2: SOM portraits of the samples studied. Samples were grouped according to the classification provided in (9, 10) (part A) and according to the novel stratification scheme suggested by us (part B).


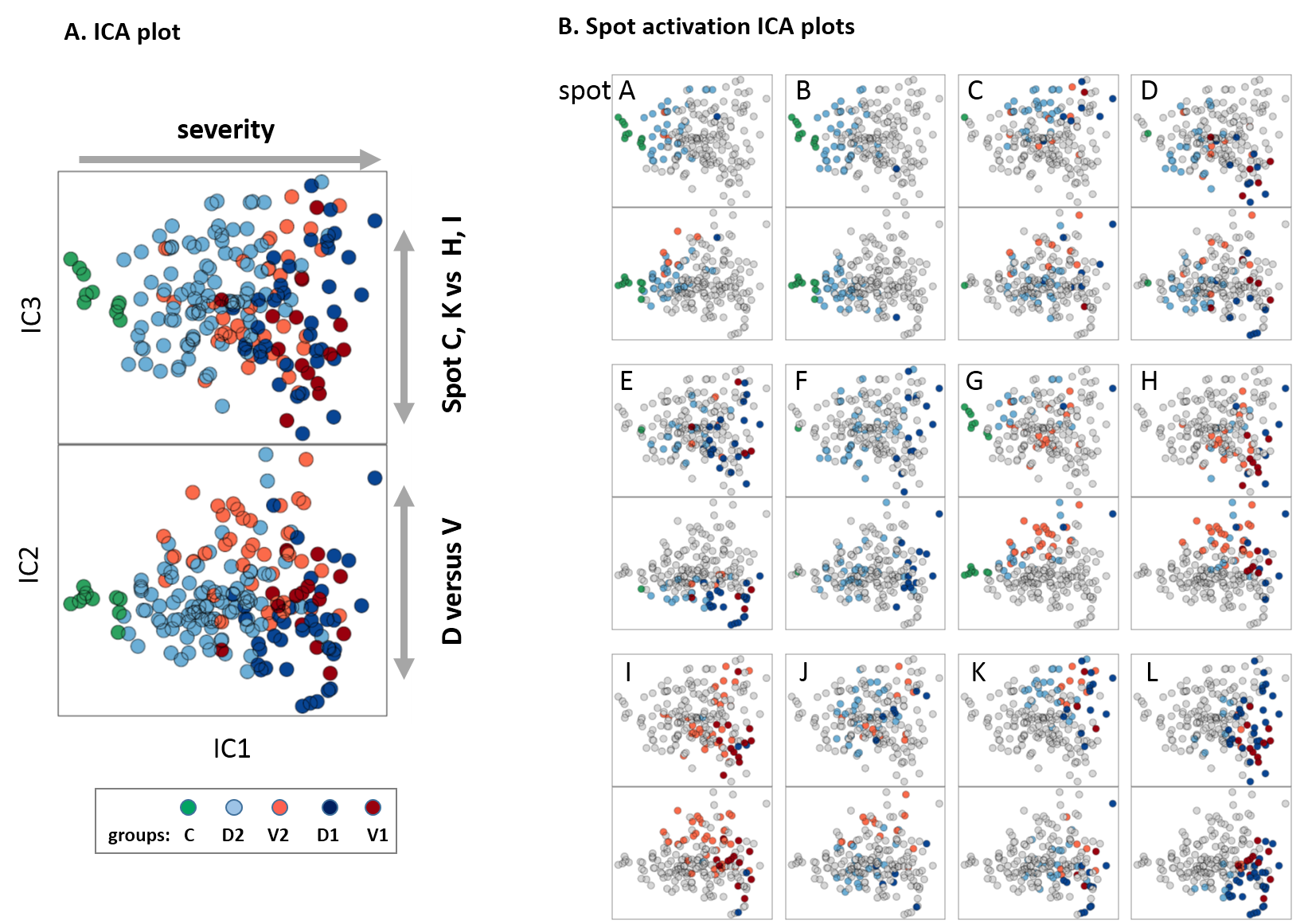


Figure S 3: Independent component analysis (ICA) of the cases studied: A) The plot of the first three independent components (IC1 – IC3) shows that IC1 describes the variability along the controls and the two CAP groups and thus the severity axis, while IC2 captures the variability introduced by the two cohorts, which differ by the amount of viral infections. B) The same plot as in part A, where however, only samples with activated spots A – L were colored in each of the plots. One sees for example that spot A is activated predominantly in the healthy controls while spot L is upregulated in group 1 severe CAP cases.


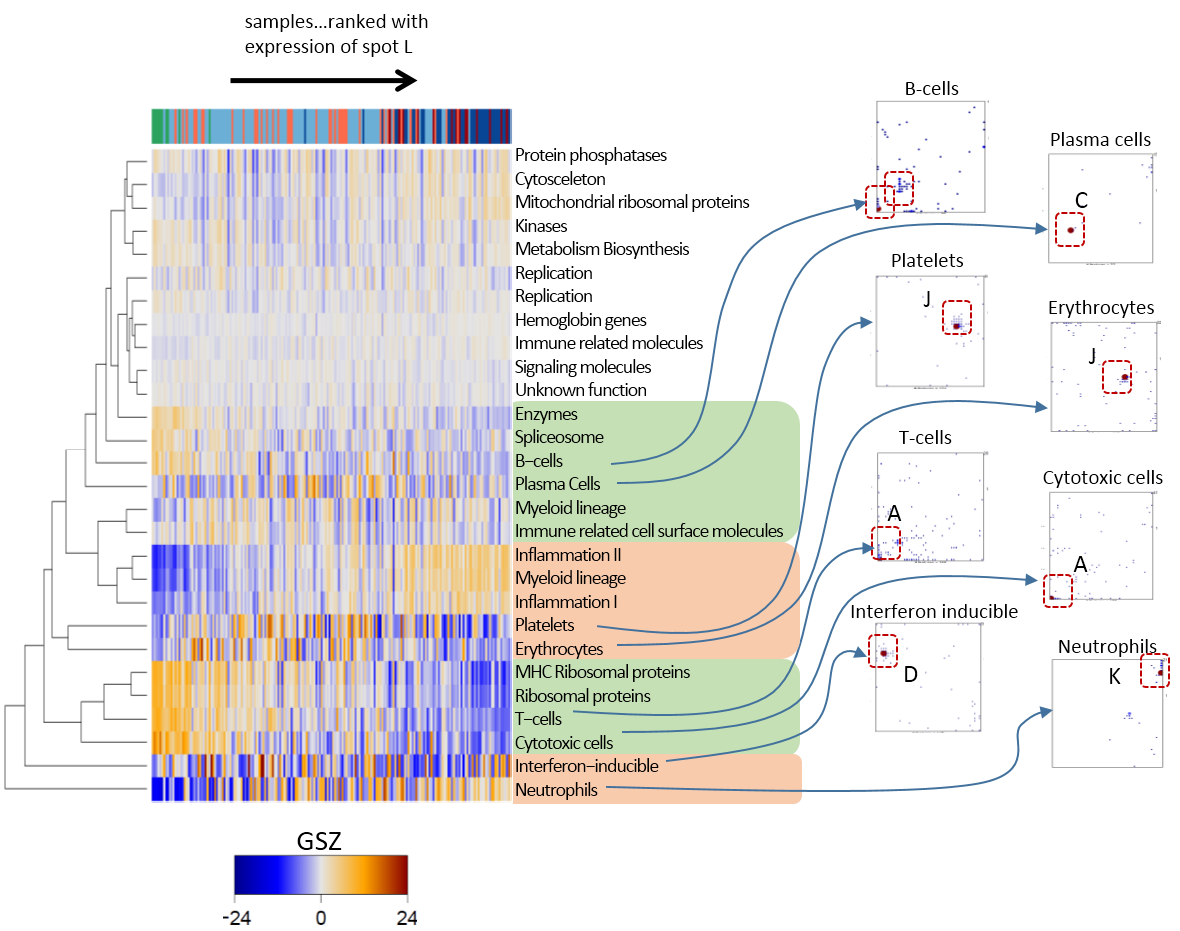


Figure S 4: GSZ-heatmap of the gene expression-modules determined in WPB in (20) in the data set studied here. Selected gene sets were mapped into the SOM where the respective genes strongly accumulate in distinct regions of the map (right part, see red frames). These areas agree with distinct spot modules (see letters and Figure 1B). Hence, the different branches of hierarchical clustering tree transform into different spots in the SOM.


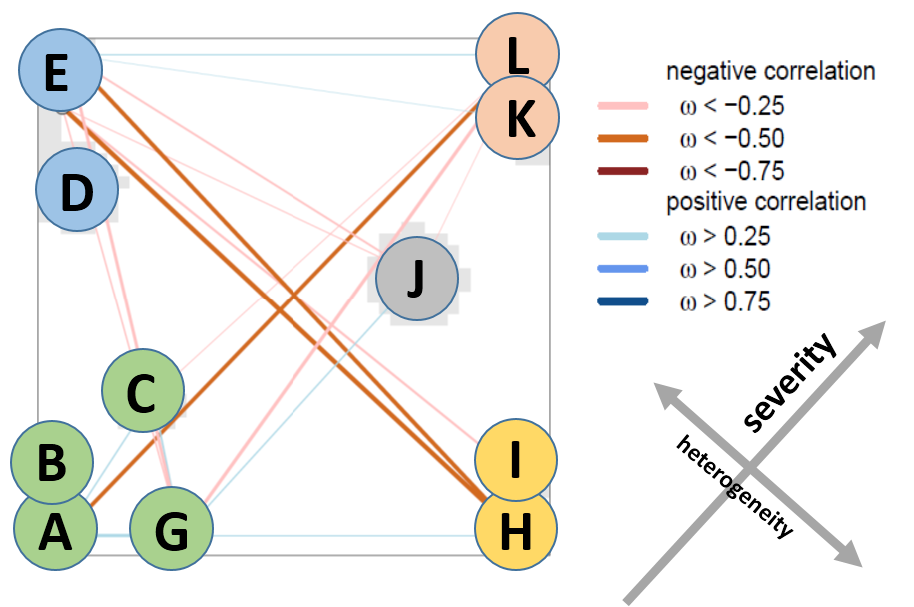


Figure S 5: Correlation structure of the SOM: Positive and negative correlations between the spots are shown by blue and read lines, respectively. The two main dimensions of heterogeneity were characterized by negative correlations between the respective spots. Correlation measures were calculated using the weighted-topology-overlap (wto) algorithm (19).


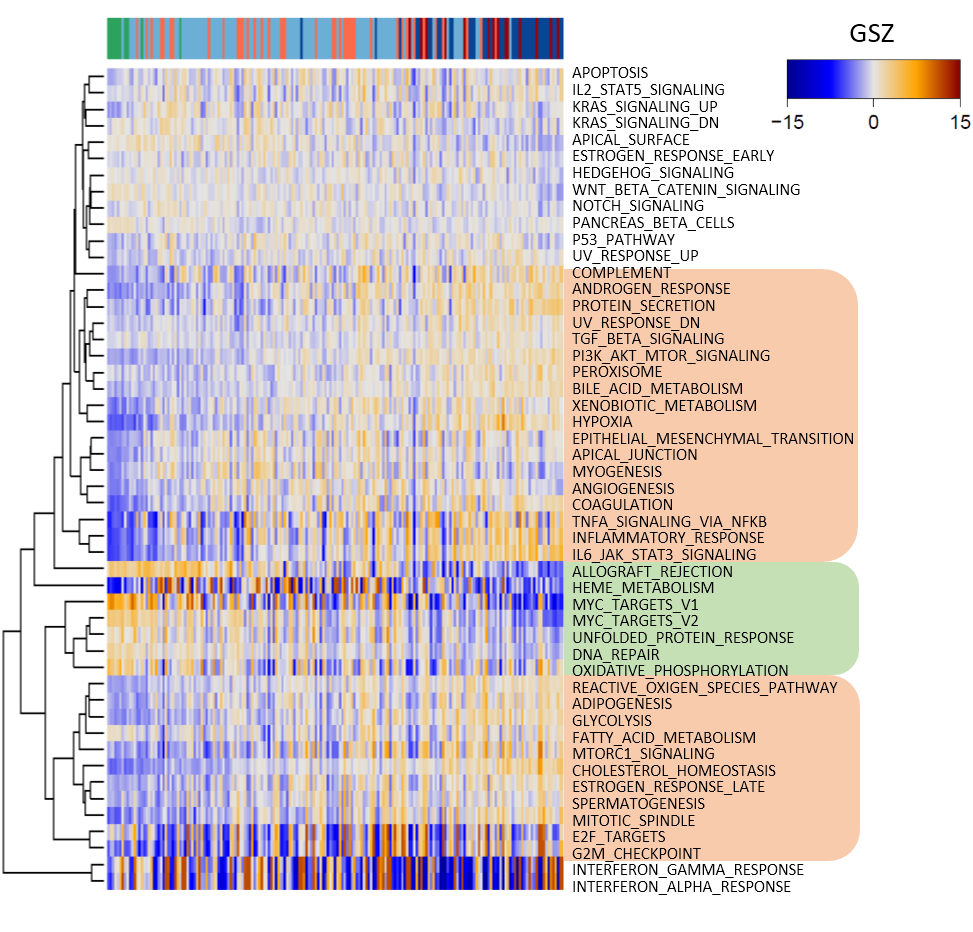


Figure S 6: Heatmap of the expression of gene sets of the category hallmarks of cancer (27). The ‘hallmarks of cancer’ gene sets characterize dysfunctional cellular programs in general, i.e. they are applicable beyond cancer biology. Most of them gain in activity in CAP because they are linked to inflammatory processes. A smaller fraction of processes mainly related to proliferation (MYC-targets), energy metabolism (oxphos) and transcription (DNA-repair) decay in CAP. A third group shows more scattered profiles and relates to erythrocytes (heme metabolism) and viral infections (interferon response).

**
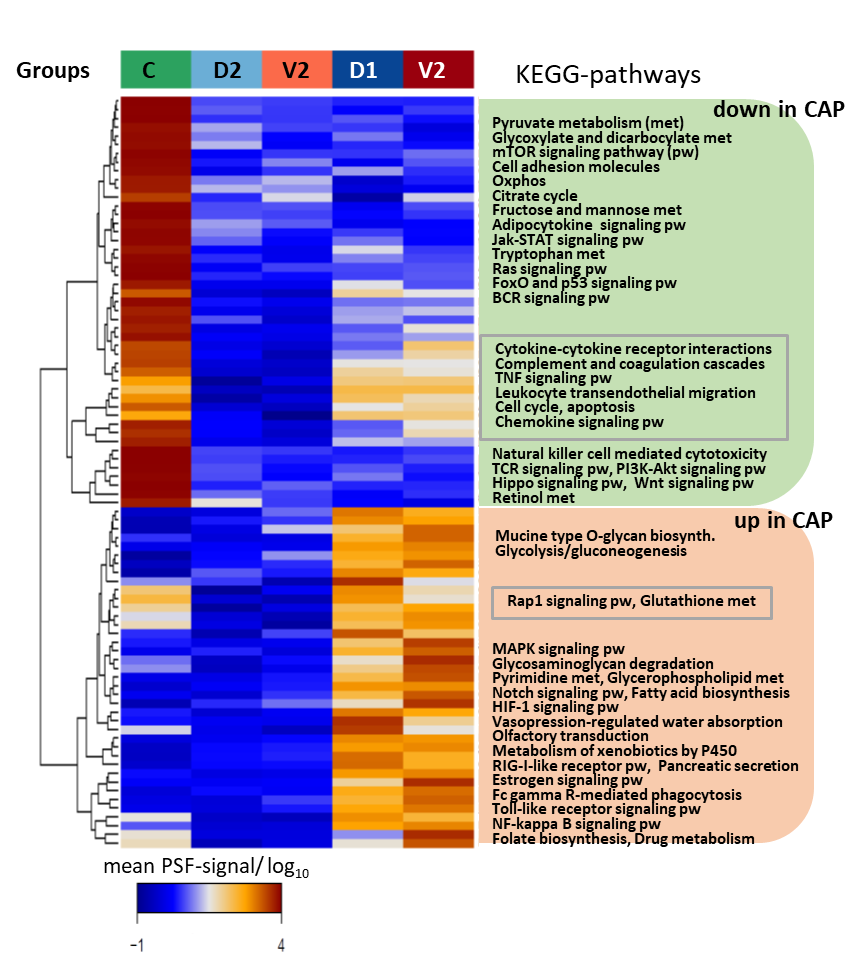
**

Figure S 7: The pathway activity heatmap divides into two major clusters of pathways of decaying activity in CAP and pathways, which become activated. The heatmap shows the mean PSF-value averaged over the genes of each pathway in all samples of each group as a measure of the mean pathway activity.


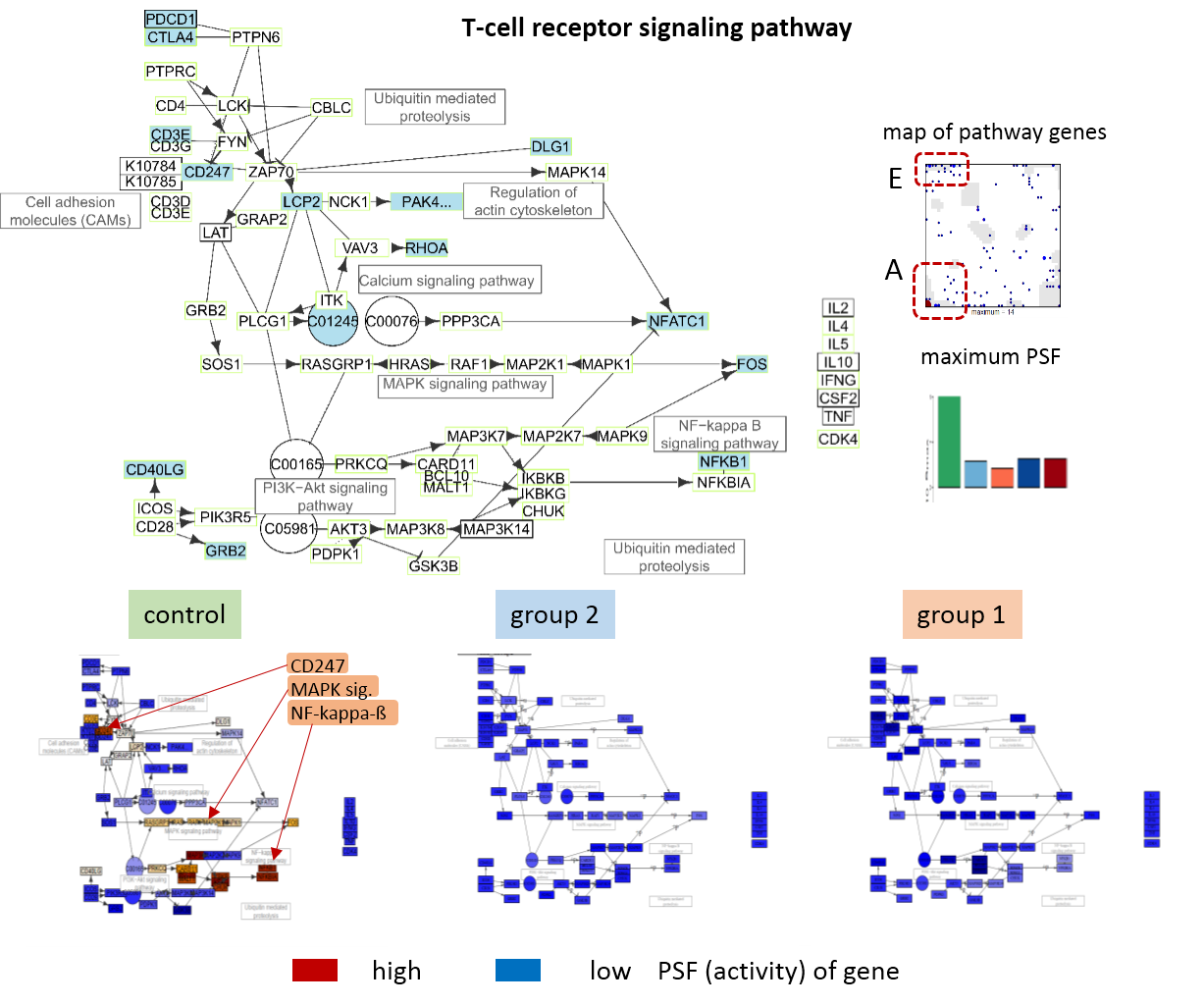


Figure S 8: Pathway signal flow (PSF) analysis of the T-cell receptor signaling pathway (TCR). The figure shows the pathway graph, the map of the pathway genes, the mean, group-averaged maximum activity of a node as boxplot in the part above. In the part below the nodes are colored according to their PSF-activity. The genes accumulate in spot A, which reflects their deactivation in CAP. First of all the call adhesion gene CD247, the NFkappaß- and MAPK-signaling branches of the pathway are active in the controls and become deactivated in CAP.


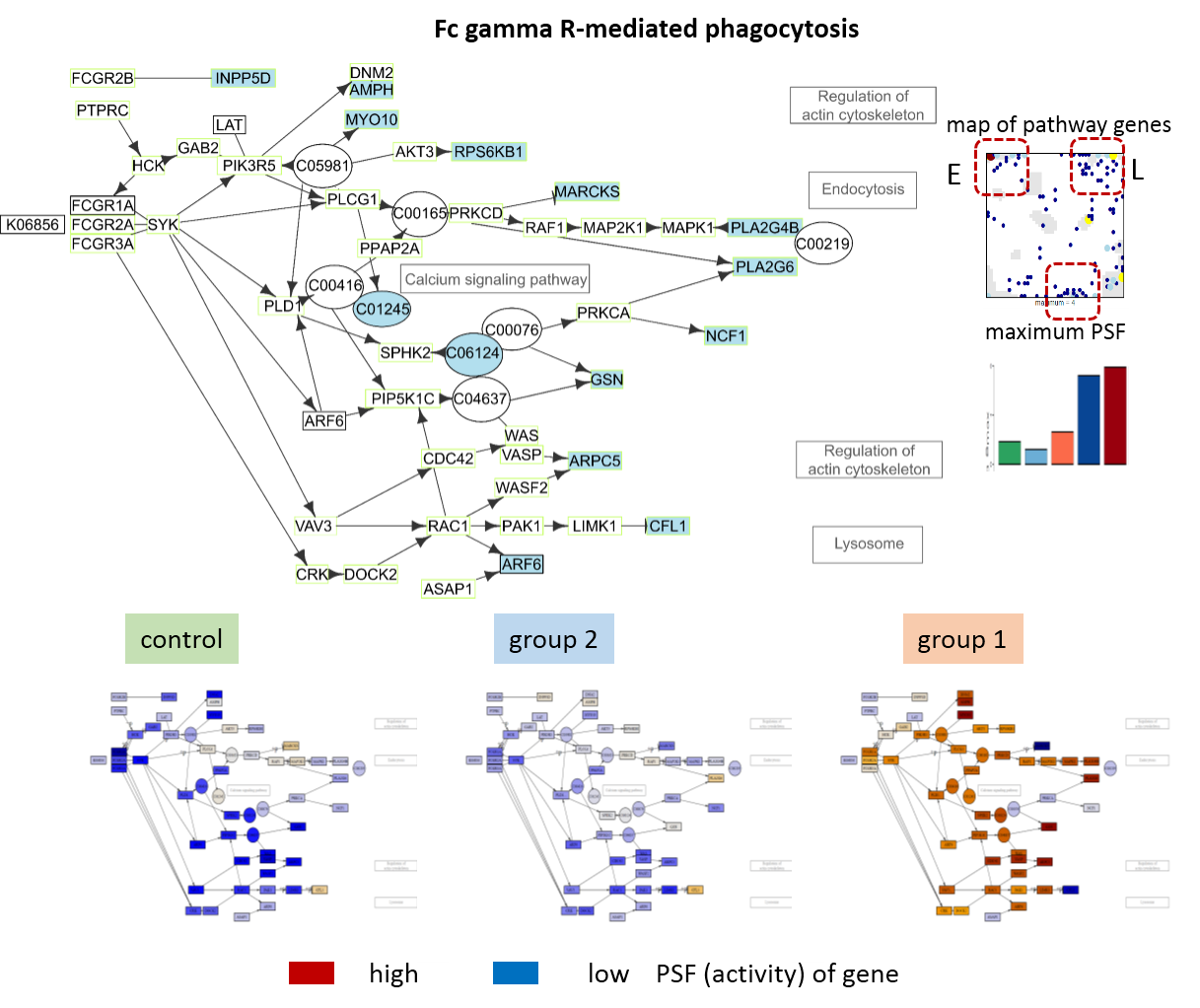


Figure S 9: Pathway signal flow (PSF) analysis of Fc-gamma R-mediated phagocytosis. See Figure S 8 for details.


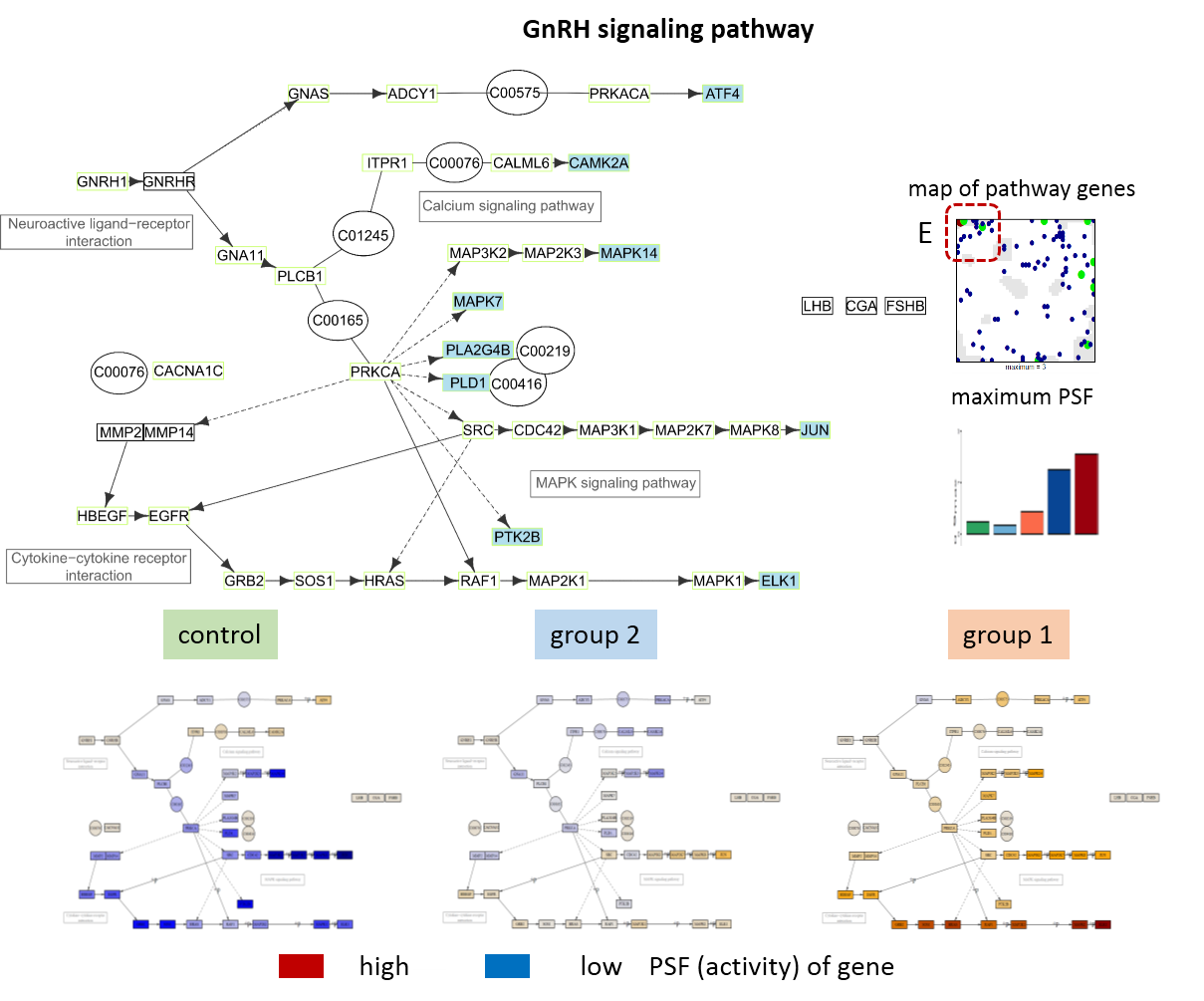


Figure S 10: Pathway signal flow (PSF) analysis of GnRH signaling pathway. See Figure S 8 for details.


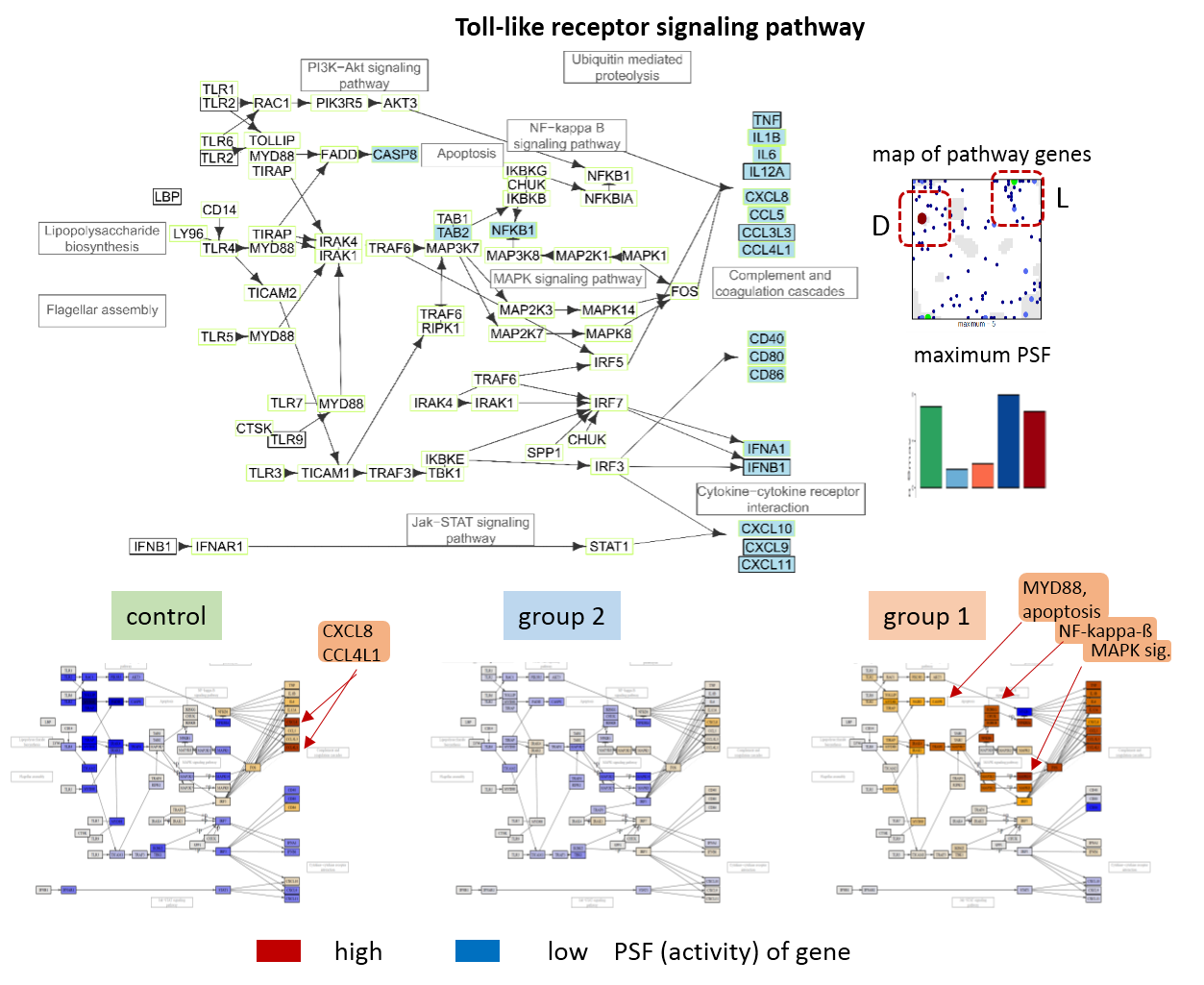


Figure S 11: Pathway signal flow (PSF) analysis of Toll-like receptor signaling pathway. See Figure S 8 for details. The MYD88-, Nfkappaß- and MAPK-signaling branches become activated in CAP.


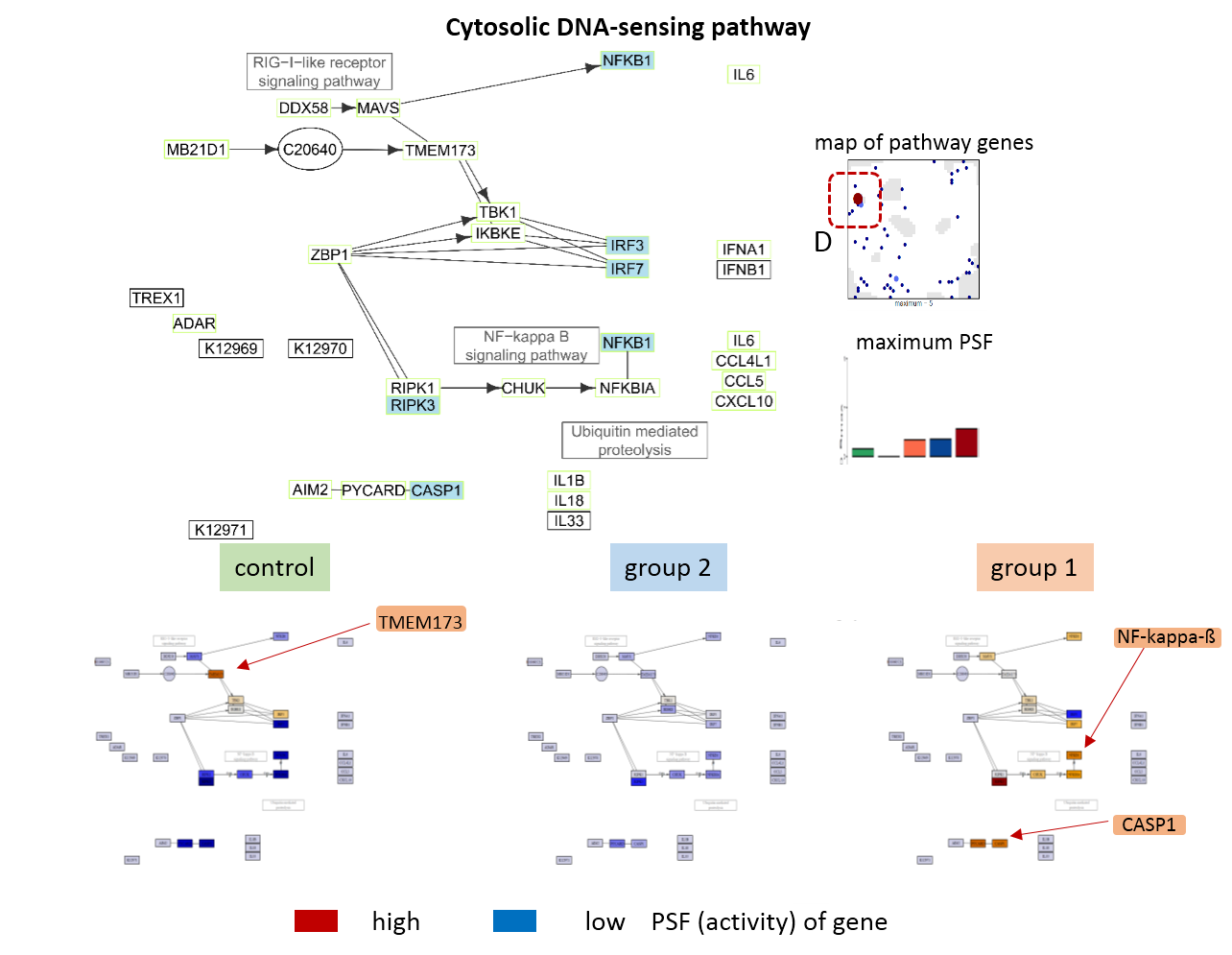


Figure S 12: Pathway signal flow (PSF) analysis of the cytosolic DNA-sensing pathway. See Figure S 8 for details.


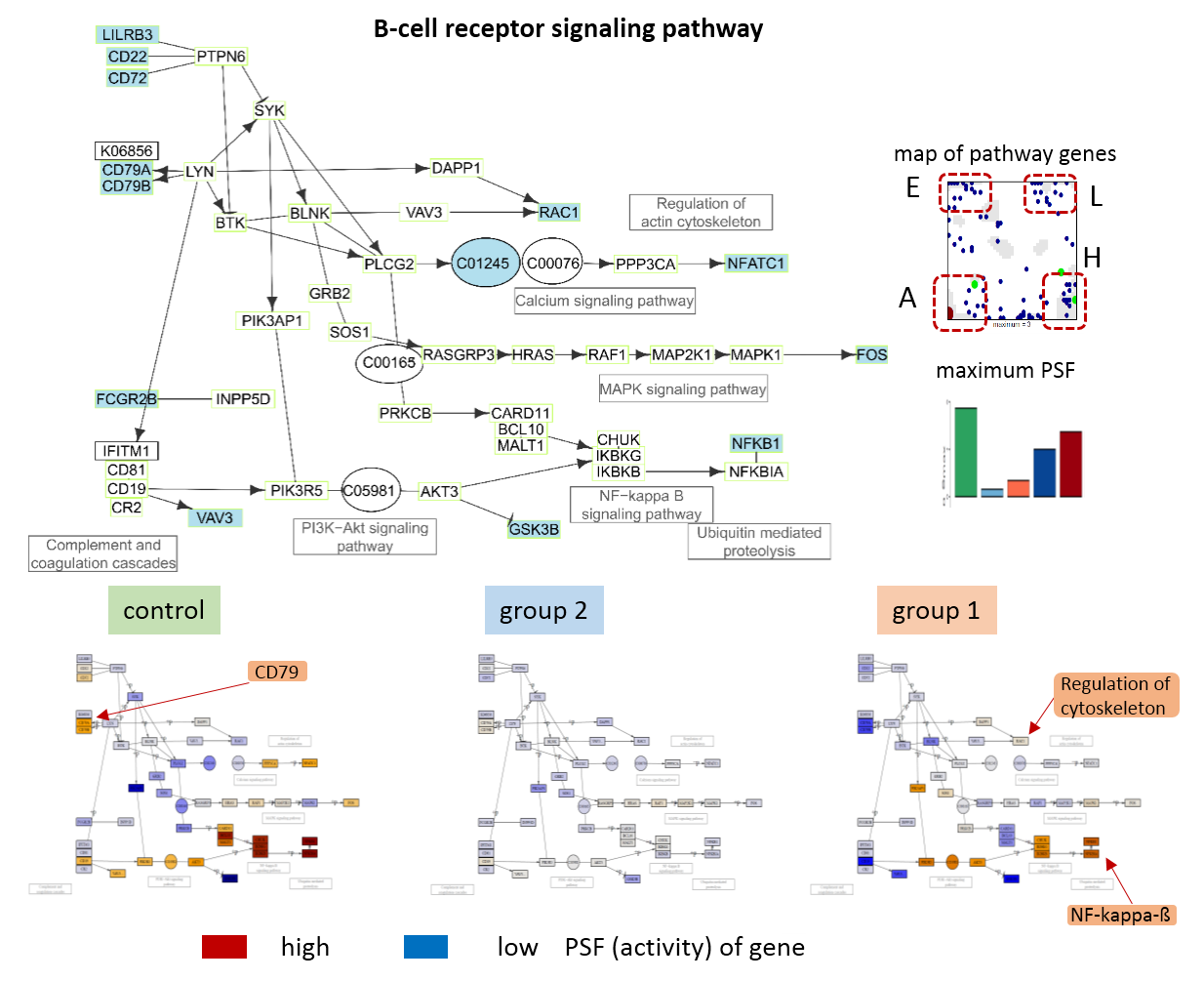


Figure S 13: Pathway signal flow (PSF) analysis of the B-cell receptor (BCR) pathway. See Figure S 8 for details.


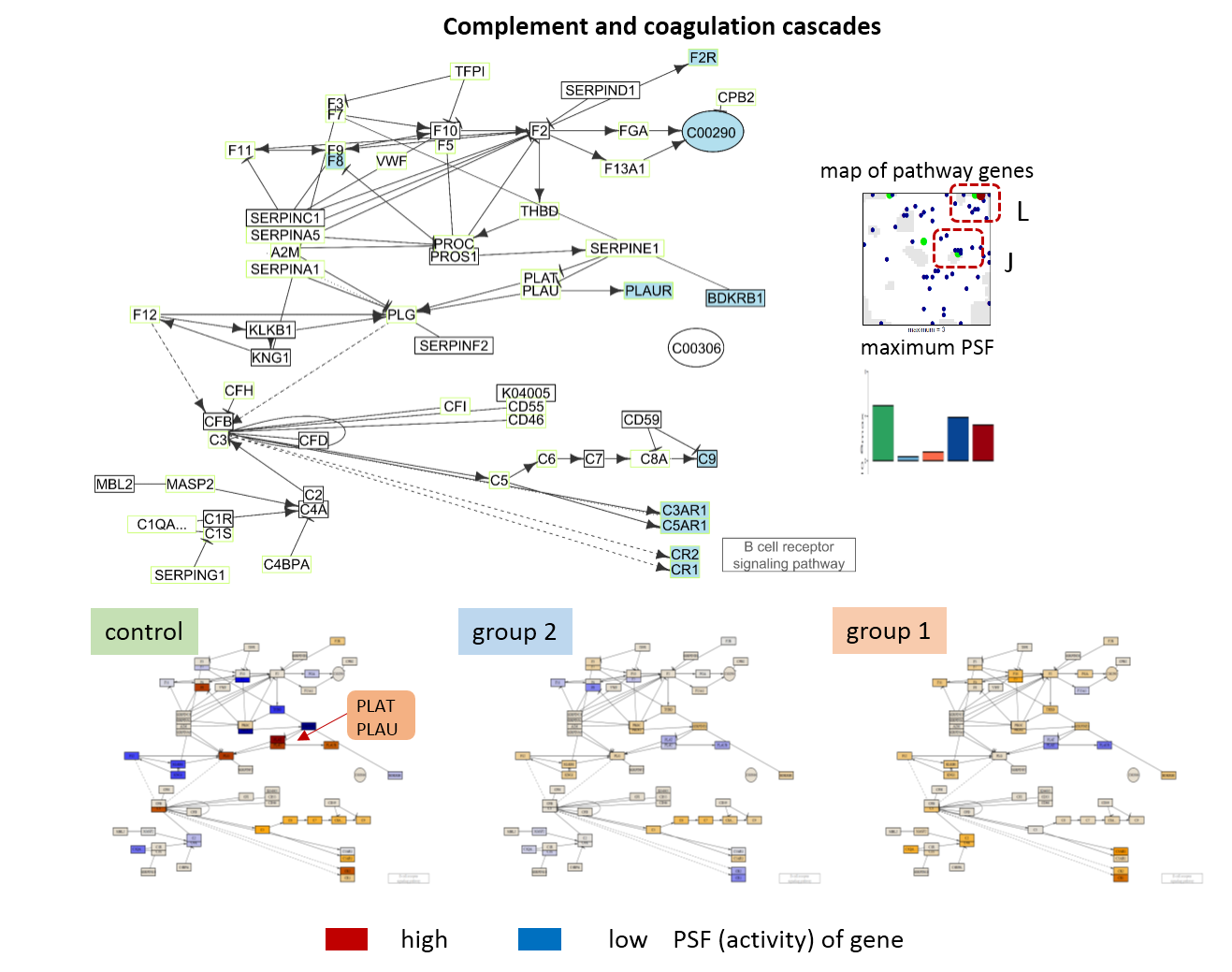


Figure S 14: Pathway signal flow (PSF) analysis of the complement and coagulation cascades pathway. See Figure S 8 for details.


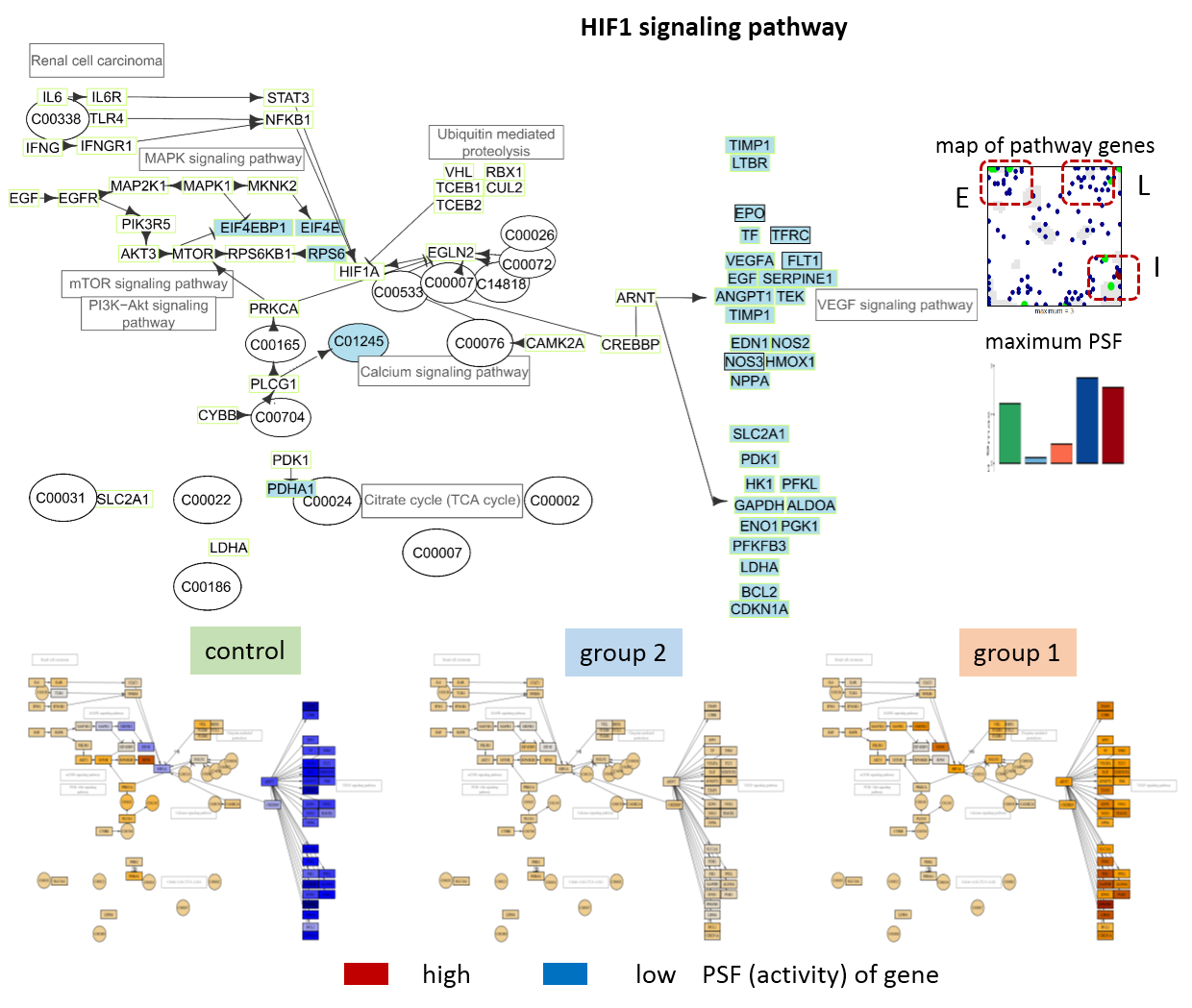


Figure S 15: Pathway signal flow (PSF) analysis of the HIF1 signaling pathway. See Figure S 8 for details.


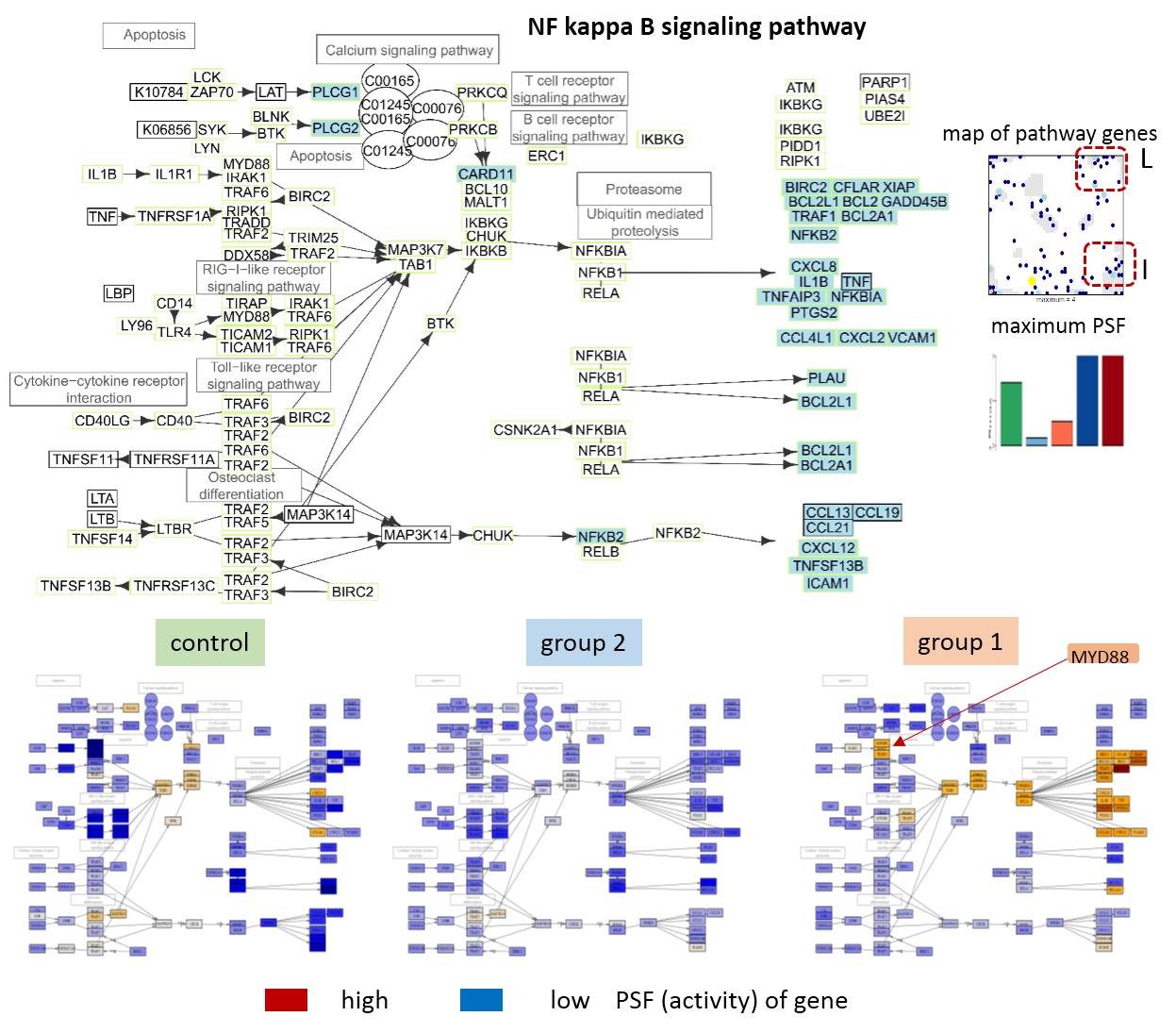


Figure S 16: Pathway signal flow (PSF) analysis of the NFkappaB pathway. See Figure S 8 for details.


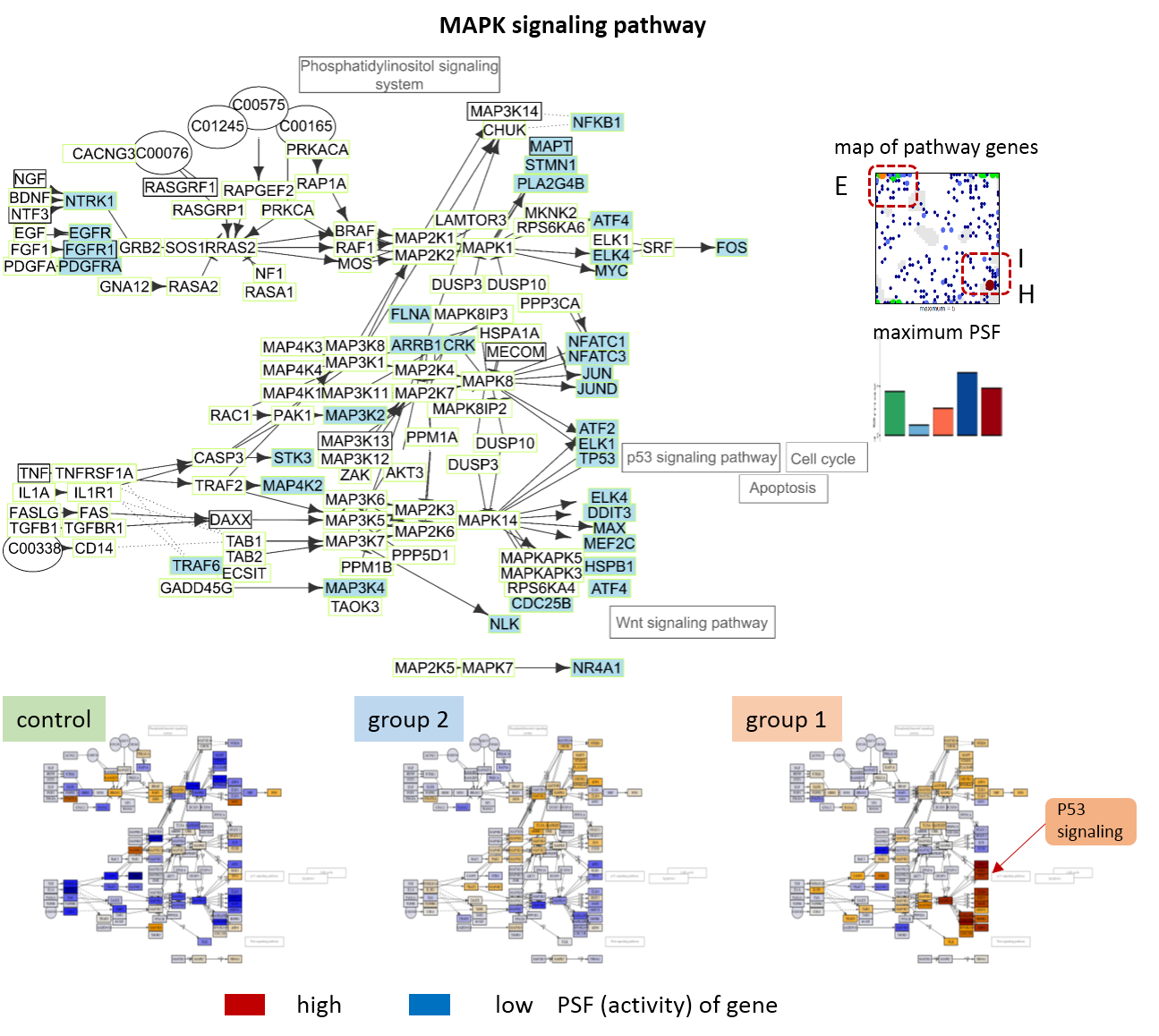


**Figure S 17**: Pathway signal flow (PSF) analysis of the MAPK signaling pathway. See **Figure S 8** for details.


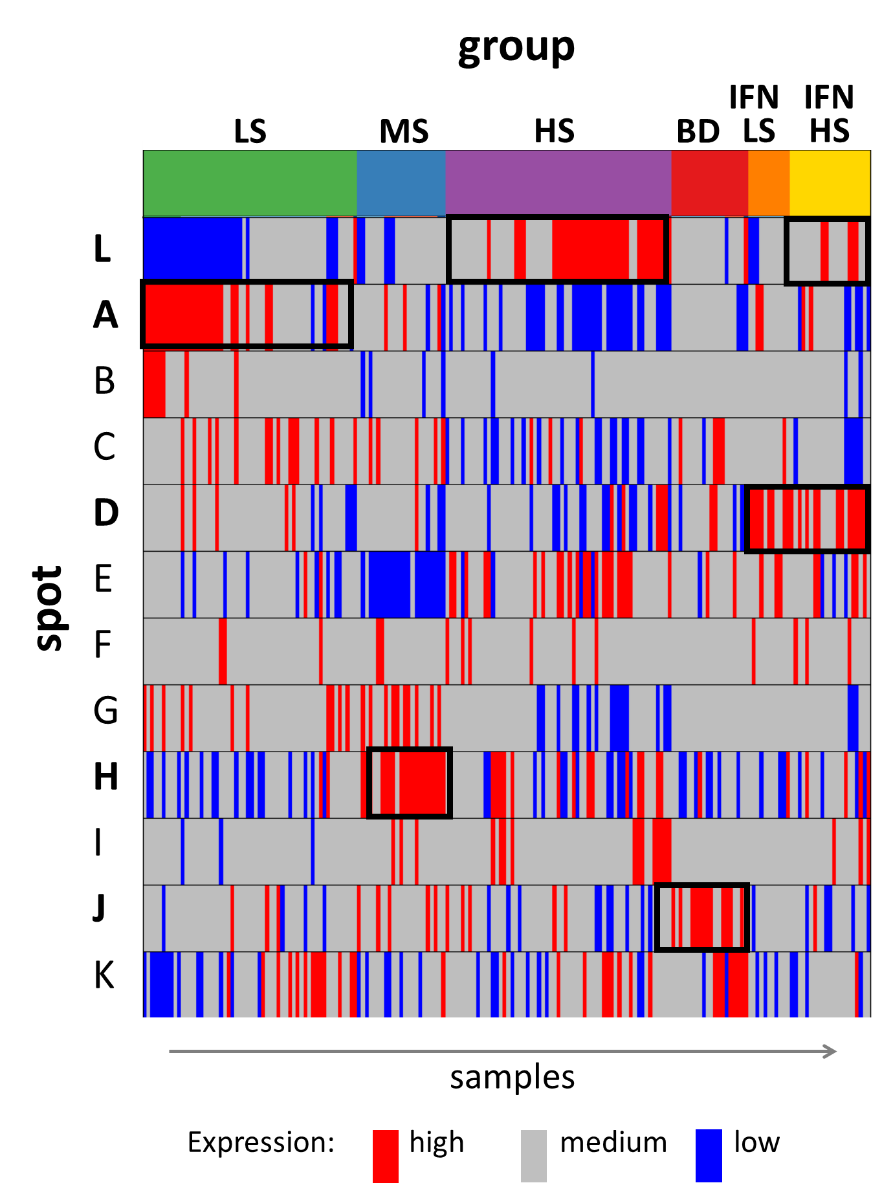


Figure S 18: Spot expression in the novel CAP groups: The expression level is coded in blue (low), grey (medium) and red (high). The groups are characterized by upregulation of spot L (HS), A (LS), D and L (IFN HS), D only (IFN LS), H (MS) and J (BD) as indicated by the black frames.


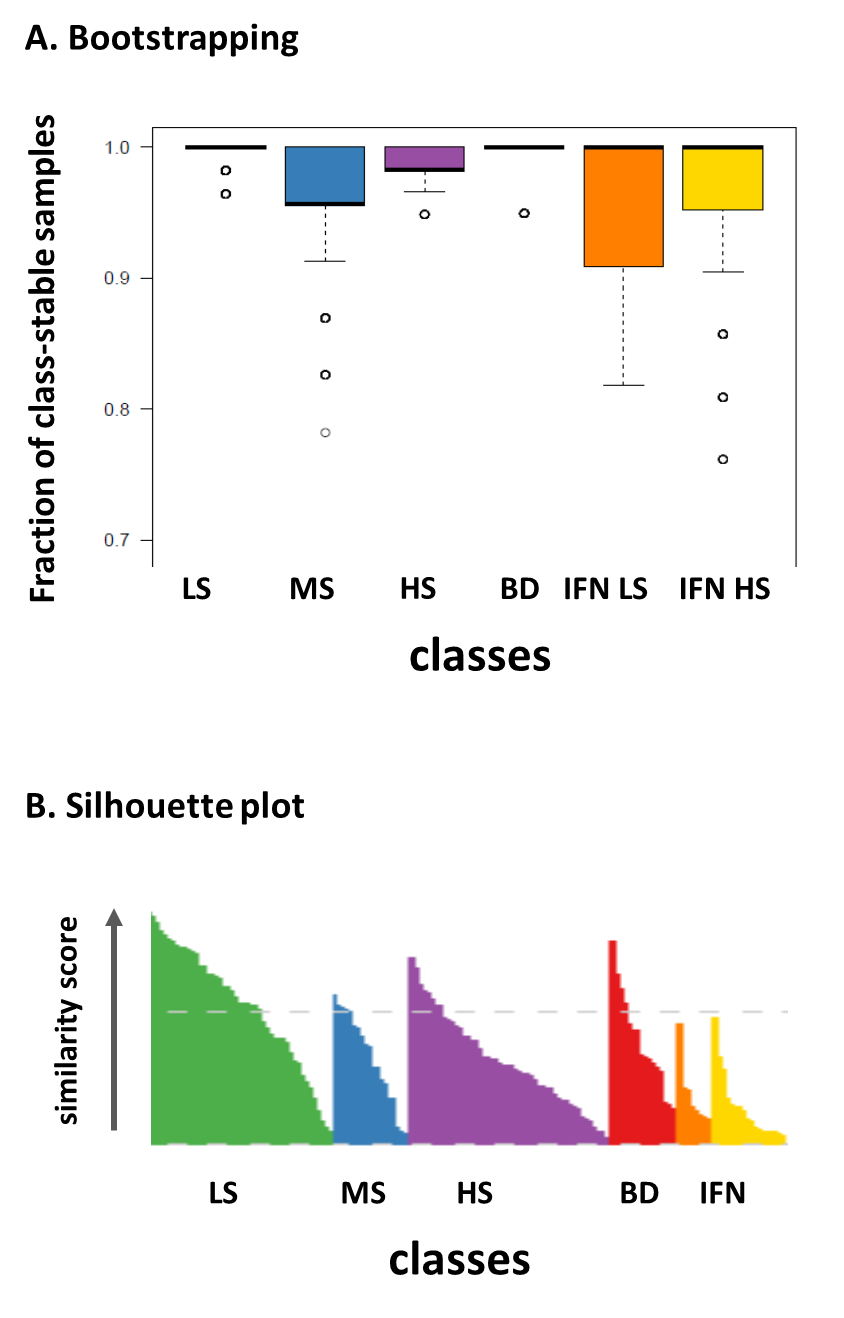


Figure S 19: Class-stability was estimated in terms of A) the fraction of class-stable samples using bootstrapping (20% leave out, 1000 repeats). More than 85% of the samples keep their class label on the average also for leave-out rates up to 40 % (data not shown). B) The silhouette plot of the novel classes reveals solely positive values for all samples thus indicating adequate clustering (compare with the silhouette plot in Figure 1D). Both methods reveal highest stability for LS and BD while IFN-LS shows lowest stability.


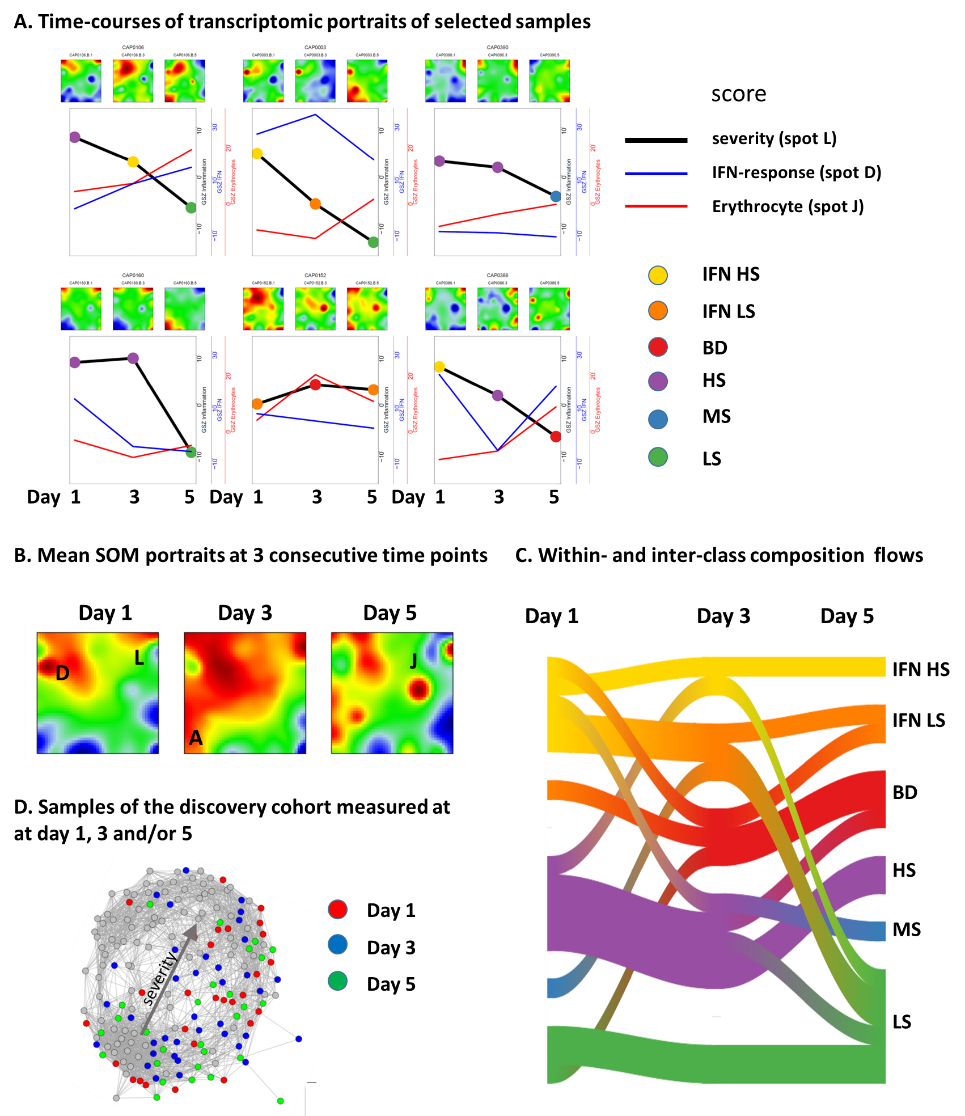


Figure S 20: Dynamics of gene expression of sepsis due to CAP of patients with serial samples collected at day 1, 3 and 5 after admission to ICU: A) Individual transcriptome portraits of selected serial samples and time courses of their transcriptomic severity, IFN-response and erythrocyte scores. The severity of CAP decays in most cases while the erythrocyte score increases in part of the samples possibly because of enhanced oxygen requirements. B) Mean SOM portraits of samples measured at day 1, 3 and 5 indicate high expression of the IFN- and high severity-related spots D and L at day 1, of spots A (low severity), E and K at day 3 and of spot J (erythrocytes) at day 5. C) Flow (Sankey-) plot of the class composition of serial samples at day 1, 3 and 5. The amount of patients increases in the low severity classes LS and IFN-LS at day 5 indicating partial recovery from sepsis while the number of patients in BD gains at day 3 and 5 possibly due to respiratory problems. D) The distribution of samples in the sample similarity net shows no clear trend except the accumulation near the low severity end at day 5. Note that time course data of the same patient are collected only in the discovery cohort that occupies mostly the right part of the network.

Table S 1: Gene sets of different functional categories used in this publication

Attached as Excel table.

Table S 2: Lists of genes of spot-modules A – L

Attached as Excel table.

Table S 3: Stratification of patient characteristics according to the novel CAP groups

| **group** | **Number of samples** | **28-days survival, % ^a^** | **Age ^a^** | **Sex ^b^**  **f m** | |
| --- | --- | --- | --- | --- | --- |
| **Low severity LS** | 46 | 89 | 59±17 | 18 | 28 |
| **Medium severity MS** | 23 | 78 | 67±13 | 4 | 19 |
| **High severity HS** | 59 | 76 | 65±12 | 30 | 29 |
| **Blood disturbances BD** | 20 | 80 | 68±17 | 9 | 11 |
| **IFN LS** | 11 | 100 | 71±15 | 5 | 6 |
| **IFN HS** | 21 | 62 | 58±19 | 13 | 8 |

**enrichment analysis (red arrays: *p* < 0.05, grey arrays: 0.05 < *p* < 0.1)**:

^a^ survival and age: two-sided Wilcoxon test

^b^ sex (f/m…female/male): Fisher’s exact test

Table S 4: Stratification of the 28-days survival rate and of the number of samples of the novel CAP groups with respect to the discovery and validation cohorts.

| **Cohort** | **Discovery** | | **Validation** | |
| --- | --- | --- | --- | --- |
| **group** | **28-days survival, %** | Number of samples | **28-days survival, %** | Number of samples |
| **Low severity LS** | **87** | 39 | **100** | 7 |
| **Medium severity MS** | **56** | 9 | **93** | 14 |
| **High severity HS** | **69** | 39 | **90** | 20 |
| **Blood disturbances BD** | **80** | 15 | **80** | 5 |
| **IFN LS** | **100** | 10 | **100** | 1 |
| **IFN HS** | **67** | 15 | **50** | 6 |

1. Morgan AJ, Glossop AJ. Severe community-acquired pneumonia. *BJA Education* (2016) 16(5):167-72. doi: 10.1093/bjaed/mkv052.

2. Pereira JM, Paiva JA, Rello J. Severe sepsis in community-acquired pneumonia &#x2014; Early recognition and treatment. *European Journal of Internal Medicine* (2012) 23(5):412-9. doi: 10.1016/j.ejim.2012.04.016.

3. Seligman R, Ramos-Lima LF, do Amaral Oliveira V, Sanvicente C, Pacheco EF, Rosa KD. Biomarkers in community-acquired pneumonia: A state-of-the-art review. *Clinics* (2012) 67(11):1321-5. doi: 10.6061/clinics/2012(11)17. PubMed PMID: PMC3488993.

4. Marti C, Garin N, Grosgurin O, Poncet A, Combescure C, Carballo S, et al. Prediction of severe community-acquired pneumonia: a systematic review and meta-analysis. *Critical Care* (2012) 16(4):R141. doi: 10.1186/cc11447.

5. Viasus D, Simonetti A, Garcia-Vidal C, Carratalà J. Prediction of prognosis by markers in community-acquired pneumonia. *Expert Review of Anti-infective Therapy* (2013) 11(9):917-29. doi: 10.1586/14787210.2013.825442.

6. Khan F, Owens MB, Restrepo M, Povoa P, Martin-Loeches I. Tools for outcome prediction in patients with community acquired pneumonia. *Expert Review of Clinical Pharmacology* (2017) 10(2):201-11. doi: 10.1080/17512433.2017.1268051.

7. Uhlén M, Hallström BM, Lindskog C, Mardinoglu A, Pontén F, Nielsen J. Transcriptomics resources of human tissues and organs. *Molecular Systems Biology* (2016) 12(4):862-n/a. doi: 10.15252/msb.20155865.

8. Mohr S, Liew C-C. The peripheral-blood transcriptome: new insights into disease and risk assessment. *Trends in Molecular Medicine* (2007) 13(10):422-32. doi: 10.1016/j.molmed.2007.08.003.

9. Davenport EE, Burnham KL, Radhakrishnan J, Humburg P, Hutton P, Mills TC, et al. Genomic landscape of the individual host response and outcomes in sepsis: a prospective cohort study. *The Lancet Respiratory Medicine* (2016) 4(4):259-71. doi: 10.1016/s2213-2600(16)00046-1.

10. Burnham KL, Davenport EE, Radhakrishnan J, Humburg P, Gordon AC, Hutton P, et al. Shared and Distinct Aspects of the Sepsis Transcriptomic Response to Fecal Peritonitis and Pneumonia. *American Journal of Respiratory and Critical Care Medicine* (2017) 196(3):328-39. doi: 10.1164/rccm.201608-1685OC. PubMed PMID: 28036233.

11. Scicluna BP, van Vught LA, Zwinderman AH, Wiewel MA, Davenport EE, Burnham KL, et al. Classification of patients with sepsis according to blood genomic endotype: a prospective cohort study. *The Lancet Respiratory Medicine* (2017) 5(10):816-26. doi: 10.1016/s2213-2600(17)30294-1.

12. Wirth H, Löffler M, von Bergen M, Binder H. Expression cartography of human tissues using self organizing maps. *BMC Bioinformatics* (2011) 12(1):306. doi: 10.1186/1471-2105-12-306.

13. Binder H, Wirth H. Analysis of large-scale OMIC data using Self Organizing Maps. (2014. In: *Encyclopedia of Information Science and Technology Third Edition* [Internet]. IGI global; [1642-54].

14. Camp JG, Sekine K, Gerber T, Loeffler-Wirth H, Binder H, Gac M, et al. Multilineage communication regulates human liver bud development from pluripotency. *Nature* (2017) 546(7659):533-8. doi: 10.1038/nature22796

http://www.nature.com/nature/journal/v546/n7659/abs/nature22796.html#supplementary-information.

15. Binder H, Hopp L, Schweiger MR, Hoffmann S, Jühling F, Kerick M, et al. Genomic and transcriptomic heterogeneity of colorectal tumours arising in Lynch syndrome. *The Journal of Pathology* (2017) 243(2):242-54. doi: 10.1002/path.4948.

16. Gerber T, Willscher E, Loeffler-Wirth H, Hopp L, Schadendorf D, Schartl M, et al. Mapping heterogeneity in patient-derived melanoma cultures by single-cell RNA-seq. *Oncotarget* (2016) 8(1):846-62.

17. Hopp L, Willscher E, Wirth-Loeffler H, Binder H. Function Shapes Content: DNA-Methylation Marker Genes and their Impact for Molecular Mechanisms of Glioma. *Journal of Cancer Research Updates* (2015) 4(4):127-48. doi: http://dx.doi.org/10.6000/1929-2279.2015.04.04.1.

18. Binder H, Hopp L, Lembcke K, Wirth H. Personalized Disease Phenotypes from Massive OMICs Data. In: Baoying W, Ruowang L, William P, editors. *Big Data Analytics in Bioinformatics and Healthcare*. Hershey, PA, USA: IGI Global (2015). p. 359-78.

19. Hopp L, Wirth H, Fasold M, Binder H. Portraying the expression landscapes of cancer subtypes: A glioblastoma multiforme and prostate cancer case study. *Systems Biomedicine* (2013) 1(2):99-121.

20. Chaussabel D, Quinn C, Shen J, Patel P, Glaser C, Baldwin N, et al. A Modular Analysis Framework for Blood Genomics Studies: Application to Systemic Lupus Erythematosus. *Immunity* (2008) 29(1):150-64. doi: 10.1016/j.immuni.2008.05.012.

21. Gimenez JLG, Carbonell NE, Mateo CR, López EG, Palacios L, Chova LP, et al. Epigenetics As The Driving Force In Long-Term Immunosuppression. *J of Clinical Epigenetics* (2016) 2(2).

22. Vucic EA, Thu KL, Robison K, Rybaczyk LA, Chari R, Alvarez CE, et al. Translating cancer ‘omics’ to improved outcomes. *Genome Res* (2012) 22(2):188-95. doi: 10.1101/gr.124354.111.

23. Wirth H, von Bergen M, Binder H. Mining SOM expression portraits: Feature selection and integrating concepts of molecular function *BioData Mining* (2012) 5:18.

24. Löffler-Wirth H, Kalcher M, Binder H. oposSOM: R-package for high-dimensional portraying of genome-wide expression landscapes on bioconductor. *Bioinformatics* (2015) 31(19):3225-7. doi: 10.1093/bioinformatics/btv342.

25. Toronen P, Ojala P, Marttinen P, Holm L. Robust extraction of functional signals from gene set analysis using a generalized threshold free scoring function. *BMC Bioinformatics* (2009) 10(1):307. PubMed PMID: doi:10.1186/1471-2105-10-307.

26. Subramanian A, Tamayo P, Mootha VK, Mukherjee S, Ebert BL, Gillette MA, et al. Gene set enrichment analysis: A knowledge-based approach for interpreting genome-wide expression profiles. *Proceedings Of The National Academy Of Sciences Of The United States Of America* (2005) 102(43):15545-50.

27. Liberzon A, Birger C, Thorvaldsdóttir H, Ghandi M, Mesirov Jill P, Tamayo P. The Molecular Signatures Database Hallmark Gene Set Collection. *Cell Systems* (2015) 1(6):417-25.

28. Roadmap Epigenomics Consortium, Kundaje A, Meuleman W, Ernst J, Bilenky M, Yen A, et al. Integrative analysis of 111 reference human epigenomes. *Nature* (2015) 518(7539):317-30. doi: 10.1038/nature14248

http://www.nature.com/nature/journal/v518/n7539/abs/nature14248.html#supplementary-information.

29. Kunz M, Löffler-Wirth H, Dannemann M, Willscher E, Dose G, Kelso J, et al. RNA-seq analysis identifies different transcriptomic types and developmental trajectories of primary melanomas. *Oncogene* (2017) under review.

30. Nersisyan L, Loeffler-Wirth H, Arakelyan A, Binder H. Gene set- and pathway- centered knowledge discovery assigns transcriptional activation patterns in brain, blood and colon cancer - A bioinformatics perspective. *Journal of Bioinformatics Knowledge Mining* (2016) 4(2):46-70.

31. Jongeneel CV, Delorenzi M, Iseli C, Zhou D, Haudenschild CD, Khrebtukova I, et al. An atlas of human gene expression from massively parallel signature sequencing (MPSS). *Genome Res* (2005) 15(7):1007-14. doi: 10.1101/gr.4041005. PubMed PMID: PMC1172045.

32. Ben-Porath I, Thomson MW, Carey VJ, Ge R, Bell GW, Regev A, et al. An embryonic stem cell-like gene expression signature in poorly differentiated aggressive human tumors. *Nat Genet* (2008) 40(5):499-507. doi: http://www.nature.com/ng/journal/v40/n5/suppinfo/ng.127_S1.html.

33. Lu C, Han HD, Mangala LS, Ali-Fehmi R, Newton CS, Ozbun L, et al. Regulation of Tumor Angiogenesis by EZH2. *Cancer Cell* (2010) 18(2):185-97. doi: 10.1016/j.ccr.2010.06.016.

34. Cheng S-C, Quintin J, Cramer RA, Shepardson KM, Saeed S, Kumar V, et al. mTOR/HIF1α-mediated aerobic glycolysis as metabolic basis for trained immunity. *Science (New York, NY)* (2014) 345(6204):1250684-. doi: 10.1126/science.1250684. PubMed PMID: PMC4226238.

35. Mantovani A, Sica A, Sozzani S, Allavena P, Vecchi A, Locati M. The chemokine system in diverse forms of macrophage activation and polarization. *Trends in Immunology* (2004) 25(12):677-86. doi: 10.1016/j.it.2004.09.015.

36. Rathinam VAK, Jiang Z, Waggoner SN, Sharma S, Cole LE, Waggoner L, et al. The AIM2 inflammasome is essential for host defense against cytosolic bacteria and DNA viruses. *Nature Immunology* (2010) 11:395. doi: 10.1038/ni.1864

https://www.nature.com/articles/ni.1864#supplementary-information.

37. Angelova M, Charoentong P, Hackl H, Fischer ML, Snajder R, Krogsdam AM, et al. Characterization of the immunophenotypes and antigenomes of colorectal cancers reveals distinct tumor escape mechanisms and novel targets for immunotherapy. *Genome Biol* (2015) 16(1):1-17. doi: 10.1186/s13059-015-0620-6.

38. Bindea G, Mlecnik B, Tosolini M, Kirilovsky A, Waldner M, Obenauf AC. Spatiotemporal dynamics of intratumoral immune cells reveal the immune landscape in human cancer. *Immunity* (2013) 39:782 - 95. doi: 10.1016/j.immuni.2013.10.003.

39. Delano MJ, Scumpia PO, Weinstein JS, Coco D, Nagaraj S, Kelly-Scumpia KM, et al. MyD88-dependent expansion of an immature GR-1<sup>+</sup>CD11b<sup>+</sup> population induces T cell suppression and Th2 polarization in sepsis. *The Journal of Experimental Medicine* (2007) 204(6):1463-74. doi: 10.1084/jem.20062602.

40. Luckheeram RV, Zhou R, Verma AD, Xia B. CD4+T Cells: Differentiation and Functions. *Clinical and Developmental Immunology* (2012) 2012:12. doi: 10.1155/2012/925135.

41. Dumeaux V, Olsen KS, Nuel G, Paulssen RH, Børresen-Dale A-L, Lund E. Deciphering Normal Blood Gene Expression Variation—The NOWAC Postgenome Study. *PLoS Genet* (2010) 6(3):e1000873. doi: 10.1371/journal.pgen.1000873.

42. Peters MJ, Joehanes R, Pilling LC, Schurmann C, Conneely KN, Powell J, et al. The transcriptional landscape of age in human peripheral blood. *Nature Communications* (2015) 6:8570. doi: 10.1038/ncomms9570

https://www.nature.com/articles/ncomms9570#supplementary-information.

43. Huan T, Esko T, Peters MJ, Pilling LC, Schramm K, Schurmann C, et al. A Meta-analysis of Gene Expression Signatures of Blood Pressure and Hypertension. *PLoS Genet* (2015) 11(3):e1005035. doi: 10.1371/journal.pgen.1005035.

44. Homuth G, Wahl S, Müller C, Schurmann C, Mäder U, Blankenberg S, et al. Extensive alterations of the whole-blood transcriptome are associated with body mass index: results of an mRNA profiling study involving two large population-based cohorts. *BMC Medical Genomics* (2015) 8:65. doi: 10.1186/s12920-015-0141-x. PubMed PMID: PMC4608219.

45. Pavia AT. What is the Role of Respiratory Viruses in Community Acquired Pneumonia; What is the Best Therapy for Influenza and Other Viral Causes of CAP? *Infectious disease clinics of North America* (2013) 27(1):157-75. doi: 10.1016/j.idc.2012.11.007. PubMed PMID: PMC3572787.

46. Andres-Terre M, McGuire Helen M, Pouliot Y, Bongen E, Sweeney Timothy E, Tato Cristina M, et al. Integrated, Multi-cohort Analysis Identifies Conserved Transcriptional Signatures across Multiple Respiratory Viruses. *Immunity* (2015) 43(6):1199-211. doi: 10.1016/j.immuni.2015.11.003.

47. Sweeney TE, Wong HR, Khatri P. Robust classification of bacterial and viral infections via integrated host gene expression diagnostics. *Science Translational Medicine* (2016) 8(346):346ra91-ra91. doi: 10.1126/scitranslmed.aaf7165. PubMed PMID: PMC5348917.

48. Biswas SK, Lopez-Collazo E. Endotoxin tolerance: new mechanisms, molecules and clinical significance. *Trends in Immunology* (2009) 30(10):475-87. doi: 10.1016/j.it.2009.07.009.

49. Morris MC, Gilliam EA, Li L. Innate Immune Programing by Endotoxin and Its Pathological Consequences. *Frontiers in Immunology* (2015) 5(680). doi: 10.3389/fimmu.2014.00680.

50. Pena OM, Hancock DG, Lyle NH, Linder A, Russell JA, Xia J, et al. An Endotoxin Tolerance Signature Predicts Sepsis and Organ Dysfunction at Initial Clinical Presentation. *EBioMedicine* (2014) 1(1):64-71. doi: 10.1016/j.ebiom.2014.10.003. PubMed PMID: PMC4326653.

51. Foster SL, Hargreaves DC, Medzhitov R. Gene-specific control of inflammation by TLR-induced chromatin modifications. *Nature* (2007) 447:972. doi: 10.1038/nature05836

https://www.nature.com/articles/nature05836#supplementary-information.

52. Coates PJ, Rundle JK, Lorimore SA, Wright EG. Indirect Macrophage Responses to Ionizing Radiation: Implications for Genotype-Dependent Bystander Signaling. *Cancer Research* (2008) 68(2):450-6. doi: 10.1158/0008-5472.can-07-3050.

53. Mantovani, Alberto, Sica, Antonio, Locati M. Macrophage Polarization Comes of Age. *Immunity* (2005) 23(4):344-6. doi: 10.1016/j.immuni.2005.10.001.

54. Furuya Y, Müllbacher A. Type I IFN Exhaustion is a Host Defence Protecting Against Secondary Bacterial Infections. *Scandinavian Journal of Immunology* (2013) 78(5):395-400. doi: 10.1111/sji.12107.

55. Burk M, El-Kersh K, Saad M, Wiemken T, Ramirez J, Cavallazzi R. Viral infection in community-acquired pneumonia: a systematic review and meta-analysis. *European Respiratory Review* (2016) 25(140):178-88. doi: 10.1183/16000617.0076-2015.

56. Shu J, Zhang F, Zhang L, Wei W. G protein coupled receptors signaling pathways implicate in inflammatory and immune response of rheumatoid arthritis. *Inflammation Research* (2017) 66(5):379-87. doi: 10.1007/s00011-016-1011-5.

57. Zhang L, Shi G. Gq-Coupled Receptors in Autoimmunity. *Journal of Immunology Research* (2016) 2016:8. doi: 10.1155/2016/3969023.

58. Lu L-F, Gavin MA, Rasmussen JP, Rudensky AY. G Protein-Coupled Receptor 83 Is Dispensable for the Development and Function of Regulatory T Cells. *Molecular and Cellular Biology* (2007) 27(23):8065-72. doi: 10.1128/mcb.01075-07.

59. Polouliakh N, Nock R, Nielsen F, Kitano H. G-Protein Coupled Receptor Signaling Architecture of Mammalian Immune Cells. *PLOS one* (2009) 4(1):e4189. doi: 10.1371/journal.pone.0004189.

60. Néemeth ZH, Leibovich SJ, Deitch EA, Vizi ES, Szabó C, Haskó G. cDNA Microarray Analysis Reveals a Nuclear Factor-κB-Independent Regulation of Macrophage Function by Adenosine. *Journal of Pharmacology and Experimental Therapeutics* (2003) 306(3):1042-9. doi: 10.1124/jpet.103.052944.

61. Sweeney TE, Shidham A, Wong HR, Khatri P. A comprehensive time-course–based multicohort analysis of sepsis and sterile inflammation reveals a robust diagnostic gene set. *Science Translational Medicine* (2015) 7(287):287ra71-ra71. doi: 10.1126/scitranslmed.aaa5993.

62. Middleton EA, Weyrich AS, Zimmerman GA. Platelets in Pulmonary Immune Responses and Inflammatory Lung Diseases. *Physiological Reviews* (2016) 96(4):1211-59. doi: 10.1152/physrev.00038.2015. PubMed PMID: 27489307.

63. Kabanova S, Kleinbongard P, Volkmer J, Andrée B, Kelm M, Jax TW. Gene expression analysis of human red blood cells. *International Journal of Medical Sciences* (2009) 6(4):156-9. PubMed PMID: PMC2677714.

64. Rowley JW, Schwertz H, Weyrich AS. Platelet mRNA: the meaning behind the message. *Current Opinion in Hematology* (2012) 19(5):385-91. doi: 10.1097/MOH.0b013e328357010e. PubMed PMID: PMC3670814.

65. Schoorl M, Snijders D, Boersma WG, Bartels PCM. Temporary impairment of reticulocyte haemoglobin content in subjects with community-acquired pneumonia. *International Journal of Laboratory Hematology* (2012) 34(4):390-5. doi: 10.1111/j.1751-553X.2012.01408.x.

66. Schoorl M, Snijders D, Schoorl M, Boersma WG, Bartels PCM. Transient impairment of reticulocyte hemoglobin content and hepcidin-25 induction in patients with community-acquired pneumonia. *Scandinavian Journal of Clinical and Laboratory Investigation* (2013) 73(1):54-60. doi: 10.3109/00365513.2012.735694.

67. Reade MC, Weissfeld L, Angus DC, Kellum JA, Milbrandt EB. The prevalence of anemia and its association with 90-day mortality in hospitalized community-acquired pneumonia. *BMC Pulmonary Medicine* (2010) 10(1):15. doi: 10.1186/1471-2466-10-15.

68. ElMaraghy AA, AbdelFattah EB, Ahmed MS. Platelet count: Is it a possible marker for severity and outcome of community acquired pneumonia? *Egyptian Journal of Chest Diseases and Tuberculosis* (2016) 65(2):499-504. doi: https://doi.org/10.1016/j.ejcdt.2015.09.001.

69. Morrell CN, Aggrey AA, Chapman LM, Modjeski KL. Emerging roles for platelets as immune and inflammatory cells. *Blood* (2014) 123(18):2759-67. doi: 10.1182/blood-2013-11-462432. PubMed PMID: PMC4007605.

70. Doré LC, Crispino JD. Transcription factor networks in erythroid cell and megakaryocyte development. *Blood* (2011) 118(2):231-9. doi: 10.1182/blood-2011-04-285981. PubMed PMID: PMC3138678.

71. Iwasaki H, Mizuno S-i, Wells RA, Cantor AB, Watanabe S, Akashi K. GATA-1 Converts Lymphoid and Myelomonocytic Progenitors into the Megakaryocyte/Erythrocyte Lineages. *Immunity* (2003) 19(3):451-62. doi: https://doi.org/10.1016/S1074-7613(03)00242-5.

72. Snow JW, Orkin SH. Translational Isoforms of FOG1 Regulate GATA1-interacting Complexes. *Journal of Biological Chemistry* (2009) 284(43):29310-9. doi: 10.1074/jbc.M109.043497.

73. Vyas P, Ault K, Jackson CW, Orkin SH, Shivdasani RA. Consequences of GATA-1 Deficiency in Megakaryocytes and Platelets. *Blood* (1999) 93(9):2867-75.

74. Del Vecchio GC, Giordani L, De Santis A, De Mattia D. Dyserythropoietic Anemia and Thrombocytopenia due to a Novel Mutation in GATA-1. *Acta Haematologica* (2005) 114(2):113-6.

75. Byrnes JR, Wolberg AS. Red blood cells in thrombosis. *Blood* (2017) 130(16):1795-9. doi: 10.1182/blood-2017-03-745349.

76. Chalmers JD, Singanayagam A, Scally C, Hill AT. Admission D-dimer Can Identify Low-Risk Patients With Community-Acquired Pneumonia. *Annals of Emergency Medicine* (2008) 53(5):633-8. doi: 10.1016/j.annemergmed.2008.12.022.

77. Salluh JIF, Rabello LSCF, Rosolem MM, Soares M, Bozza FA, Verdeal JCR, et al. The impact of coagulation parameters on the outcomes of patients with severe community-acquired pneumonia requiring intensive care unit admission. *Journal of Critical Care* (2011) 26(5):496-501. doi: 10.1016/j.jcrc.2011.02.001.

78. Mills EW, Wangen J, Green R, Ingolia NT. Dynamic regulation of a ribosome rescue pathway in erythroid cells and platelets. *Cell Reports* (2016) 17(1):1-10. doi: 10.1016/j.celrep.2016.08.088. PubMed PMID: PMC5111367.

79. Scicluna BP, Klein Klouwenberg PMC, van Vught LA, Wiewel MA, Ong DSY, Zwinderman AH, et al. A Molecular Biomarker to Diagnose Community-acquired Pneumonia on Intensive Care Unit Admission. *American Journal of Respiratory and Critical Care Medicine* (2015) 192(7):826-35. doi: 10.1164/rccm.201502-0355OC.

80. Ernst J, Kellis M. Discovery and characterization of chromatin states for systematic annotation of the human genome. *Nat Biotech* (2010) 28(8):817-25. doi: http://www.nature.com/nbt/journal/v28/n8/abs/nbt.1662.html#supplementary-information.

81. Hebenstreit D, Fang M, Gu M, Charoensawan V, van Oudenaarden A, Teichmann SA. RNA sequencing reveals two major classes of gene expression levels in metazoan cells. *Mol Syst Biol* (2011) 7. doi: http://www.nature.com/msb/journal/v7/n1/suppinfo/msb201128_S1.html.

82. Ernst J, Kheradpour P, Mikkelsen TS, Shoresh N, Ward LD, Epstein CB, et al. Mapping and analysis of chromatin state dynamics in nine human cell types. *Nature* (2011) 473(7345):43-9. doi: http://www.nature.com/nature/journal/v473/n7345/abs/10.1038-nature09906-unlocked.html#supplementary-information.

83. De Santa F, Totaro MG, Prosperini E, Notarbartolo S, Testa G, Natoli G. The Histone H3 Lysine-27 Demethylase Jmjd3 Links Inflammation to Inhibition of Polycomb-Mediated Gene Silencing. *Cell* 130(6):1083-94. doi: 10.1016/j.cell.2007.08.019.

84. Bannister AJ, Kouzarides T. Regulation of chromatin by histone modifications. *Cell Research* (2011) 21(3):381-95. doi: 10.1038/cr.2011.22. PubMed PMID: PMC3193420.

85. Hopp L, Nersisyan L, Löffler-Wirth H, Arakelyan A, Binder H. Epigenetic Heterogeneity of B-Cell Lymphoma: Chromatin Modifiers. *Genes* (2015) 6(4):1076. PubMed PMID: doi:10.3390/genes6041076.

86. Rasmussen KD, Helin K. Role of TET enzymes in DNA methylation, development, and cancer. *Genes & Development* (2016) 30(7):733-50. doi: 10.1101/gad.276568.115.

87. Yu J, Qiu Y, Yang J, Bian S, Chen G, Deng M, et al. DNMT1-PPARγ pathway in macrophages regulates chronic inflammation and atherosclerosis development in mice. *Scientific Reports* (2016) 6:30053. doi: 10.1038/srep30053

https://www.nature.com/articles/srep30053#supplementary-information.

88. Cheng C, Huang C, Ma T-T, Bian E-B, He Y, Zhang L, et al. SOCS1 hypermethylation mediated by DNMT1 is associated with lipopolysaccharide-induced inflammatory cytokines in macrophages. *Toxicology Letters* (2014) 225(3):488-97. doi: https://doi.org/10.1016/j.toxlet.2013.12.023.

89. Zhou D, Yang K, Chen L, Zhang W, Xu Z, Zuo J, et al. Promising landscape for regulating macrophage polarization: epigenetic viewpoint. *Oncotarget* (2017) 8(34):57693-706. doi: 10.18632/oncotarget.17027. PubMed PMID: PMC5593677.

90. Cull AH, Snetsinger B, Buckstein R, Wells RA, Rauh MJ. Tet2 restrains inflammatory gene expression in macrophages. *Experimental Hematology* (2017) 55:56-70.e13. doi: 10.1016/j.exphem.2017.08.001.

91. Xue S, Liu C, Sun X, Li W, Zhang C, Zhou X, et al. TET3 Inhibits Type I IFN Production Independent of DNA Demethylation. *Cell Reports* (2016) 16(4):1096-105. doi: 10.1016/j.celrep.2016.06.068.

92. Brown TA, Lee JW, Holian A, Porter V, Fredriksen H, Kim M, et al. Alterations in DNA methylation corresponding with lung inflammation and as a biomarker for disease development after MWCNT exposure. *Nanotoxicology* (2016) 10(4):453-61. doi: 10.3109/17435390.2015.1078852.

93. Lopez-Pastrana J, Shao Y, Chernaya V, Wang H, Yang X-F. Epigenetic enzymes are the therapeutic targets for CD4+CD25+Foxp3+ regulatory T cells. *Translational Research* (2015) 165(1):221-40. doi: 10.1016/j.trsl.2014.08.001.

94. Ang P, Loh M, Liem N, Lim P, Grieu F, Vaithilingam A, et al. Comprehensive profiling of DNA methylation in colorectal cancer reveals subgroups with distinct clinicopathological and molecular features. *BMC Cancer* (2010) 10(1):227. PubMed PMID: doi:10.1186/1471-2407-10-227.

95. Noushmehr H, Weisenberger DJ, Diefes K, Phillips HS, Pujara K, Berman BP, et al. Identification of a CpG Island Methylator Phenotype that Defines a Distinct Subgroup of Glioma. *Cancer Cell* (2010) 17(5):510-22. doi: http://dx.doi.org/10.1016/j.ccr.2010.03.017.

96. Hopp L, Wirth-Loeffler H, Binder H. Epigenetic heterogeneity of B-cell lymphoma: DNA-methylation, gene expression and chromatin states. *Genes* (2015) 6(3):812-40. doi: doi:10.3390/genes6030812.

97. Voigt P, Tee W-W, Reinberg D. A double take on bivalent promoters. *Genes & Development* (2013) 27(12):1318-38. doi: 10.1101/gad.219626.113.

98. Rodriguez J, Muñoz M, Vives L, Frangou CG, Groudine M, Peinado MA. Bivalent domains enforce transcriptional memory of DNA methylated genes in cancer cells. *Proc Natl Acad Sci USA* (2008) 105(50):19809-14. doi: 10.1073/pnas.0810133105.

99. Arakelyan A, Nersisyan L, Poghosyan D, Khondkaryan L, Hakobyan A, Löffler-Wirth H, et al. Autoimmunity and autoinflammation: A systems view on signaling pathway dysregulation profiles. *PLOS one* (2017) 12(11):e0187572. doi: 10.1371/journal.pone.0187572.

100. Cakir MV, Binder H, Wirth H. Profiling of Genetic Switches using Boolean Implications in Expression Data. *Journal of Integrative Bioinformatics* (2014) 11(1):246. doi: 10.2390/biecoll-jib-2014-246.

101. Palsson-McDermott EM, O'Neill LAJ. The Warburg effect then and now: From cancer to inflammatory diseases. *Bioessays* (2013) 35(11):965-73. doi: 10.1002/bies.201300084.

102. Quintin J, Saeed S, Martens JHA, Giamarellos-Bourboulis EJ, Ifrim DC, Logie C, et al. Candida albicans Infection Affords Protection against Reinfection via Functional Reprogramming of Monocytes. *Cell host & microbe* (2012) 12(2):10.1016/j.chom.2012.06.006. doi: 10.1016/j.chom.2012.06.006. PubMed PMID: PMC3864037.

103. Ostuni R, Piccolo V, Barozzi I, Polletti S, Termanini A, Bonifacio S, et al. Latent Enhancers Activated by Stimulation in Differentiated Cells. *Cell* (2013) 152(1):157-71. doi: 10.1016/j.cell.2012.12.018.

104. Suarez-Alvarez B, Rodriguez RM, Fraga MF, López-Larrea C. DNA methylation: a promising landscape for immune system-related diseases. *Trends in Genetics* 28(10):506-14. doi: 10.1016/j.tig.2012.06.005.
